# Long‐Chain Acyl Carnitines Aggravate Polystyrene Nanoplastics‐Induced Atherosclerosis by Upregulating MARCO

**DOI:** 10.1002/advs.202205876

**Published:** 2023-05-05

**Authors:** Bo Wang, Boxuan Liang, Yuji Huang, Zhiming Li, Bingli Zhang, Jiaxin Du, Rongyi Ye, Hongyi Xian, Yanhong Deng, Jiancheng Xiu, Xingfen Yang, Sahoko Ichihara, Gaku Ichihara, Yizhou Zhong, Zhenlie Huang

**Affiliations:** ^1^ NMPA Key Laboratory for Safety Evaluation of Cosmetics Guangdong Provincial Key Laboratory of Tropical Disease Research School of Public Health Southern Medical University Guangzhou 510515 China; ^2^ Affiliated Dongguan People's Hospital Southern Medical University Dongguan 523059 China; ^3^ State Key Laboratory of Organ Failure Research Department of Cardiology Nanfang Hospital Southern Medical University Guangzhou 510515 China; ^4^ Department of Environmental and Preventive Medicine School of Medicine Jichi Medical University Tochigi 329‐0498 Japan; ^5^ Department of Occupational and Environmental Health Faculty of Pharmaceutical Sciences Tokyo University of Science Noda 278‐8510 Japan

**Keywords:** atherosclerosis, cardiovascular toxicity, long‐chain acyl carnitines, macrophage activation, microplastics and nanoplastics

## Abstract

Exposure to micro‐ and nanoplastics (MNPs) is common because of their omnipresence in environment. Recent studies have revealed that MNPs may cause atherosclerosis, but the underlying mechanism remains unclear. To address this bottleneck, *ApoE* ^−/−^ mice are exposed to 2.5–250 mg kg^−1^ polystyrene nanoplastics (PS‐NPs, 50 nm) by oral gavage with a high‐fat diet for 19 weeks. It is found that PS‐NPs in blood and aorta of mouse exacerbate the artery stiffness and promote atherosclerotic plaque formation. PS‐NPs activate phagocytosis of M1‐macrophage in the aorta, manifesting as upregulation of macrophage receptor with collagenous structure (MARCO). Moreover, PS‐NPs disrupt lipid metabolism and increase long‐chain acyl carnitines (LCACs). LCAC accumulation is attributed to the PS‐NP‐inhibited hepatic carnitine palmitoyltransferase 2. PS‐NPs, as well as LCACs alone, aggravate lipid accumulation via upregulating MARCO in the oxidized low‐density lipoprotein‐activated foam cells. Finally, synergistic effects of PS‐NPs and LCACs on increasing total cholesterol in foam cells are found. Overall, this study indicates that LCACs aggravate PS‐NP‐induced atherosclerosis by upregulating MARCO. This study offers new insight into the mechanisms underlying MNP‐induced cardiovascular toxicity, and highlights the combined effects of MNPs with endogenous metabolites on the cardiovascular system, which warrant further study.

## Graphical abstract

Exposure of *ApoE* ^−/−^ mice to 50 nm polystyrene nanoplastics (PS‐NPs) with a high‐fat diet explores the roles of macrophage and lipid metabolism disruption in micro‐ and nanoplastic‐induced atherosclerosis. Results indicate that long‐chain acyl carnitines (LCACs) aggravate PS‐NP‐induced atherosclerosis by upregulating macrophage receptor with collagenous structure, and highlights the synergistic effects of PS‐NPs with endogenous metabolite LCACs on the cardiovascular system.

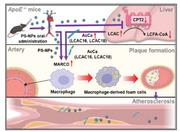

## 1 Introduction

Micro‐ and nanoplastics (MNPs) are emerging contaminants due to their persistent presence in air, land, and water, which has become a significant global concern.^[^ ^1^, ^2^ ^]^ Microplastics (MPs) have been found in human blood,^[^ ^3^ ^]^ placenta,^[^ ^4^ ^]^ stool,^[^ ^5^ ^]^ meconium,^[^ ^2^ ^]^ lungs,^[^ ^1^ ^]^ and lung ground glass nodules.^[^ ^6^ ^]^ MPs can be fragmented into nanoplastics (NPs) under the influence of ultraviolet radiation, weathering, and/or biodegradation. NPs are considered a worse “extension” of MPs due to their greater capacity to penetrate into the body.^[^ ^7^ ^]^ NPs can cause brain toxicity,^[^ ^8^ ^]^ nephrotoxicity,^[^ ^9^ ^]^ hepatotoxicity,^[^ ^10^ ^]^ pulmonary toxicity,^[^ ^11^ ^]^ intestinal toxicity,^[^ ^12^ ^]^ testicular toxicity,^[^ ^13^ ^]^ and cardiovascular toxicity.^[^ ^14^ ^]^ However, there is a large knowledge gap regarding the cardiovascular toxicity of NPs.

Atherosclerosis is a chronic inflammatory disease of large and medium‐sized arteries that causes ischemic heart disease, strokes, and peripheral vascular disease collectively called cardiovascular diseases.^[^ ^15^ ^]^ The infiltration and accumulation of plasma lipoproteins and leukocyte subsets is a driving force behind atherosclerotic lesion.^[^ ^16^, ^17^ ^]^ Indeed, macrophages play key roles in the progression of atherosclerosis, and macrophage‐associated pathological processes have been considered important targets for both diagnostic imaging and novel therapies for atherosclerosis.^[^ ^18^ ^]^ In recent studies, multiomics analysis has revealed that MNP exposure induced size‐dependent toxicity and vascular endothelial cell injury both in vivo and in vitro.^[^ ^19^, ^20^ ^]^ However in those studies, the mice were exposed to NPs by subcutaneous injection with 100 mg kg^−1^ of 20 nm polystyrene NPs (PS‐NPs).^[^ ^19^, ^20^ ^]^Those studies are detached from reality in that the MNP exposure routes and levels they examined were relevant to neither humans nor the environment in which we live. In addition, investigation into the mechanisms of PS‐NP‐induced vascular endothelial cell toxicity have been unsatisfactory.^[^ ^19^ ^]^ Currently, sulfate‐modified PS‐NPs can induce intracellular lipid accumulation and lead to the differentiation of macrophage into foam cell, a characteristic feature usually observed in the pathogenesis of atherosclerosis.^[^ ^21^ ^]^ Therefore, the key roles of macrophages in the process of NP‐induced atherosclerosis warrant further study, particularly regarding routes and levels pertinent to humans and our environment.

Lipid metabolism disruption plays a pivotal role in the onset and development of atherosclerosis.^[^ ^22^ ^]^ Serum phosphatidylcholine (PC) and lysophosphatidylcholine (LPC) profiles are altered in atherosclerotic patients.^[^ ^23^, ^24^ ^]^ Additionally, cholesterol esters, glycerophospholipids, glycerolipids, or sphingolipid metabolism disruption contributes to the development of atherosclerosis in mice.^[^ ^23^, ^24^ ^]^ Elevated plasma cholesterol is sufficient to drive the genesis of atherosclerosis, even in the absence of other known risk factors.^[^ ^25^ ^]^ Nanoparticles, e.g., carbon black, silica, and iron oxide, can alter serum or plasma lipids in vivo.^[^ ^26^, ^27^, ^28^ ^]^ Moreover, fine particulate matter (PM_2.5_) can disrupt lipid metabolism leading to atherosclerosis, and increase atherosclerotic plaque in *ApoE*‐deficient mice.^[^ ^24^ ^]^ PS‐MPs can cause changes in atherosclerosis‐associated gene expression in the perivascular adipose tissue of mouse.^[^ ^29^ ^]^ Furthermore, MPs can cause lipid metabolism disruption in vivo.^[^ ^30^, ^31^, ^32^ ^]^ Thus, the role of changes in lipid metabolism in the occurrence and evolution of NP‐induced atherosclerosis needs further investigation.

To examine the role of macrophages and lipid metabolism disruption in NP‐induced atherosclerosis, we exposed *ApoE* knockout (*ApoE*‐KO, *ApoE* ^−/−^) mice to 50 nm PS‐NPs by daily oral gavage with a high‐fat diet (HFD) for 19 consecutive weeks. We used doses ranging from 2.5 to 250 mg kg^−1^ body weight (BW), which covered real‐world human and environmental MNP levels, for both risk assessment and hazard identification. We evaluated the PS‐NP‐induced cardiovascular toxicity by multiple approaches, including blood pressure measurement, ultrasound biomicroscopy (UBM), histopathological examination, and transcriptome. Then, we applied biochemistry and lipidome analysis on the mouse plasma to investigate lipid profile changes after PS‐NP exposure. Furthermore, we verified and investigated the differential gene expression and lipid profiles altered by the PS‐NP exposure in the macrophages cotreated with oxidized low‐density lipoprotein (ox‐LDL). This study will contribute to the field of environmental science by elucidating the mechanisms of PS‐NP‐aggravated cardiovascular damage. The results of this study will help in managing the growing health risk of NP exposure posed to human beings.

## 2 Results

### 2.1 Characteristics of PS‐NP Particles

**Figure 1:**
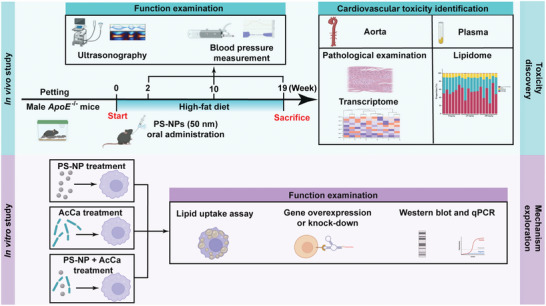
Flow chart diagram of the present study.

The strategy of the present study is presented in a flow chart diagram (**Figure** **1**). We evaluated the characteristics of PS‐NPs in food by simulating gastrointestinal digestion in vitro and in vivo (Table S1, Supporting Information). We also evaluated the characteristics of PS‐NPs in the mouse blood (Table S1, Supporting Information). The parameters included hydrodynamic size, polymer dispersity index (PDI), and zeta potential. Under the circumstance of simulating gastrointestinal digestion in vitro, three major nutrients (carbohydrates, fats, and proteins) alone could integrate with PS‐NPs (500 µg mL^−1^) and thereafter became agglomerated, which was manifested by a slight increase in hydrodynamic size, PDI, and zeta potential. When the three major nutrients coexisted, PS‐NPs were agglomerated and no monodisperse PS‐NPs were detected, evidenced by the increased hydrodynamic diameter, PDI and zeta potential. After entering the gastrointestinal tract by gavage, PS‐NPs (250 mg kg^−1^) were aggregated in the mouse stomachs, characterized with elevated hydrodynamic diameter and PDI. Interestingly, we found that PS‐NPs were agglomerated in the blood of mice, presented as increased hydrodynamic diameter and PDI.

### 2.2 PS‐NPs Distributed in the Blood, Aorta, and Liver in the *ApoE* ^−/−^ Mice

**Figure 2:**
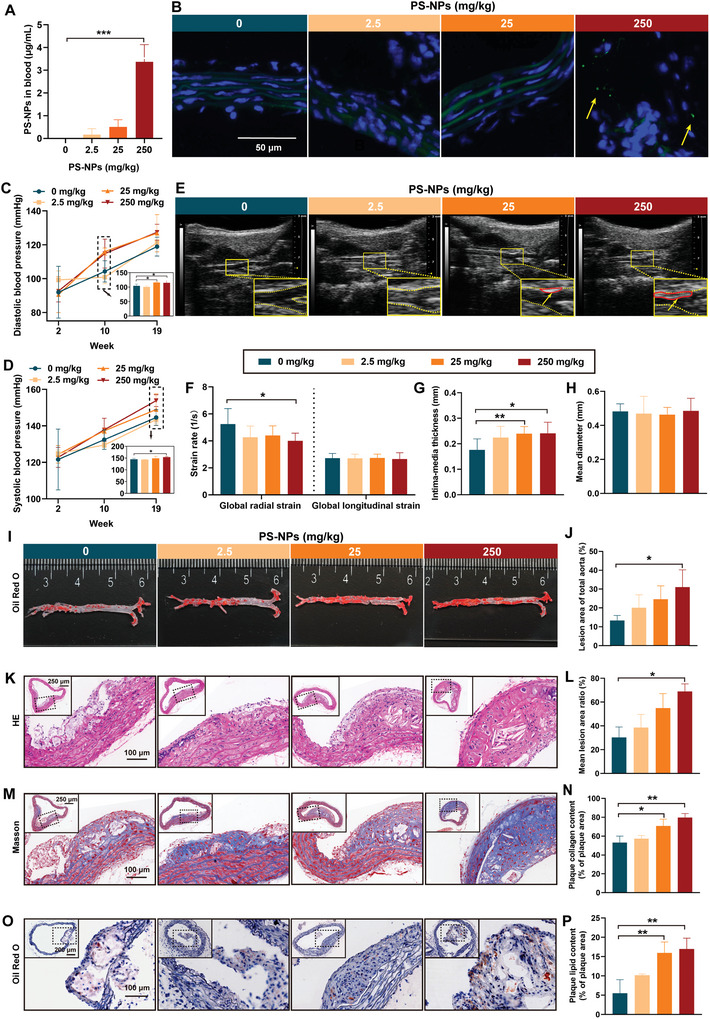
PS‐NPs exacerbated the artery stiffness and promoted formation of atherosclerotic plaque in the *ApoE* ^−/−^ mice. A) PS‐NP particles distributed in the mouse blood in a dose‐dependent manner after 24 h exposure. B) Dose‐dependent increases in the intensity of green fluorescence PS‐NP particles observed in the aortic tissue slides in the mice after 24 h PS‐NP exposure. C) Increased DBP and D) SBP during 19‐week exposure to nonfluorescence PS‐NPs. E) Ultrasound biomicroscopy on the mouse. The red circles indicated by the yellow arrows showed the image of the plaque in the left carotid artery of mice captured by ultrasound. F) A decreased global radial strain rate and G) increased IMT, and H) no change in mean diameter after 19‐week PS‐NP exposure. I,J) Plaque formation in the aortic arch and abdominal aorta in the *ApoE* ^−/−^ mice treated with HFD for 19 weeks; it was especially noticeable in the 250 mg kg^−1^ PS‐NP group. K,L) HE staining showed the mean lesion area ratio increased, and cholesterol crystals and calcification accumulated inside the aortic plaque in the 250 mg kg^−1^ PS‐NP group. M,N) Masson staining revealed increased mouse plaque collagen content in the 25 and 250 mg kg^−1^ PS‐NPs. O,P) Oil Red O staining revealed an increase in lipid depositions inside the plaque after 25 and 250 mg kg^−1^ PS‐NP exposure. Results are shown as mean ± SD. Comparisons were made with ANOVA, followed by Tukey's method. *P < 0.05, **P < 0.01, and ***P < 0.001, versus the control group. DBP: diastolic blood pressure; IMT: intima‐media thickness; LCCA: left common carotid artery; SBP: systolic blood pressure.

The PS‐NP biodistribution and its accumulation in mouse blood were investigated under single exposure with various concentrations. The standard curve for blood PS‐NP particle green fluorescence intensity was linearly correlated with its concentrations (Figure S1, Supporting Information). The PS‐NP particles were distributed in the peripheral blood in a dose‐dependent manner after 24 h exposure. The blood PS‐NP levels of the three exposed groups were 0.17, 0.51, and 3.37 µg mL^−1^, respectively (**Figure** **2**A). In light of the 1.6 µg mL^−1^ MNPs detected in human blood,^[^ ^3^ ^]^ the present PS‐NP exposure levels by oral administration in the mice were relevant to human exposure levels. Then, the histopathological observation confirmed the biodistribution of green fluorescence PS‐NPs in the aorta (Figure 2B) and liver (Figure S5, Supporting Information) tissue slides, and the intensity of particles increased dose‐dependently. These results demonstrated that the *ApoE* ^−/−^ mice had been exposed to PS‐NPs.

### 2.3 PS‐NPs Exacerbated Artery Stiffness in the *ApoE* ^−/−^ Mice

We evaluated the effect of PS‐NPs on atherosclerosis with an in vivo model, which was achieved by daily gavage of PS‐NPs on *ApoE* ^−/−^ mice fed with HFD. We measured diastolic blood pressure (DBP) and systolic blood pressure (SBP) to clarify the adverse effects of PS‐NPs on blood vessels. DBP and SBP increased during the 19‐week PS‐NP exposure (Figure 2C,D). Also, 25 and 250 mg kg^−1^ PS‐NPs significantly increased the DBP at 10‐week exposure (Figure 2C), and 250 mg kg^−1^ PS‐NPs significantly increased the SBP at 19‐week exposure (Figure 2D). We applied UBM to the mouse left common carotid artery (LCCA), a noninvasive, accurate, and inexpensive tool for the dynamic characterization of blood vessels in animal models to monitor the vascular morphology and elasticity.^[^ ^33^ ^]^ The global radial strain rate declined in all PS‐NP groups, and significantly decreased in the 250 mg kg^−1^ group (Figure 2E,F). There was no significant change in the global longitudinal strain rate after 19‐week PS‐NP exposure (Figure 2E,F). In addition, PS‐NPs increased the LCCAs intima‐media thickness (IMT) in both the 25 and the 250 mg kg^−1^ exposure groups (Figure 2E,G). However, there was no significant difference in the LCCA diameter after 19‐week PS‐NP exposure (Figure 2E,H). Overall, PS‐NP exposure exacerbated vascular stiffness in the *ApoE* ^−/−^ mice, even at the dose of 25 mg kg^−1^.

### 2.4 PS‐NPs Promoted Atherosclerotic Plaque Formation in the *ApoE* ^−/−^ Mice

The distribution and progression of lesions in the aorta were assessed through en‐face staining of the whole aorta and histopathological analysis of plaque in the aortic arch. It was found that plaque formed in the aortic arch and abdominal aorta of all the mice (Figure 2I). There was an increase in the distribution of plaque throughout the entire aorta after 250 mg kg^−1^ PS‐NP exposure compared to the control (Figure 2I,J).

Aortic arch atherosclerosis is an important source of embolic stroke.^[^ ^34^ ^]^ Thus, we performed histopathological analysis on plaque in the aortic arch to describe the characteristic components and pathogenic mechanisms. Hematoxylin–eosin (HE) staining showed that the mean lesion area ratio increased significantly in the 250 mg kg^−1^ PS‐NP group, accompanied by accumulated cholesterol crystals and calcification inside the plaque (Figure 2K,L). Masson staining revealed an increase in plaque collagen content in the mice exposed to 25 and 250 mg kg^−1^ PS‐NPs (Figure 2M,N). Oil Red O staining revealed an increase in lipid deposits inside the plaque after 25 and 250 mg kg^−1^ PS‐NP exposures (Figure 2O,P).

The features of aortas in the control group were aggregation of foam cells with a small amount of collagen fibers and matrix, and occasional cholesterol crystals in the vascular endothelium and subendothelial (Figure 2K,M,O). However, the aortas in the 250 mg kg^−1^ PS‐NP group were characterized by increased lipid pools and necrotic cores, as well as widespread cholesterol crystallization (Figure 2K,M,O). The lipid core is usually surrounded by a thick layer of fibrous connective tissue and calcification with necrosis visible in the plaque.^[^ ^35^ ^]^ To evaluate different progress stages of atherosclerosis, we analyzed the atherosclerotic lesion types according to the definition and standards reported by the American Heart Association from 1992 to 1995.^[^ ^36^, ^37^, ^38^ ^]^ We showed the pathological grades of PS‐NP‐induced aortic atherosclerosis in Figure S2 of the Supporting Information. There was considerably more pathological damage in the PS‐NP‐exposed mice than in the control mice (**Table** **1**). This result demonstrated that exposure to PS‐NPs accelerates the pathological progression of atherosclerotic plaque.

**Table 1:** Pathological grading of aortic atherosclerosis in *ApoE* ^−/−^ mice after PS‐NP exposure

Dose mg kg^−1^]	Pathological grading^a)^ (*n*)						Mean rank	*P*‐value
	I	II (Figure S2A, Supporting Information)	III (Figure S2B, Supporting Information)	IV (Figure S2C, Supporting Information)	V (Figure S2D, Supporting Information)	VI (Figure S2E, Supporting Information)		
0	0	2	3	0	0	0	2.88	–^b)^
2.5	0	0	1	2	0	1	10.00^*^	<0.05
25	0	0	1	1	2	0	10.38^*^	<0.05
250	0	0	0	0	4	1	12.00^*^	<0.05

^a)^ I: Lipid deposits, small, isolated foam cells; II: More distinct lesions, foam cells in adjacent layers, intimal smooth muscle cells contain lipid droplets; III: Extracellular lipid droplets and particles, adaptive intimal thickening, lipid pools; IV: Lipid core, severe intimal disorganization, calcium deposition; V: Multilayered lesions, have several lipid cores, hematomas or thrombi, a fibrotic layer, calcified lesions, increased fibrous connective tissue, while lipids are minimal or even absent; VI: Fissures and ulcerations in the lesion surface; thrombi or the remnants of thrombi; localized outward bulges or aneurysms, layered mural thrombi;

^b)^ Not available. One aorta sample was missing from both the 2.5 and the 25 mg kg^−1^, due to unexpected death of mice during the 19‐week PS‐NP exposure.

### 2.5 PS‐NPs Activated Macrophage Phagocytosis in the *ApoE* ^−/−^ Mice

**Figure 3:**
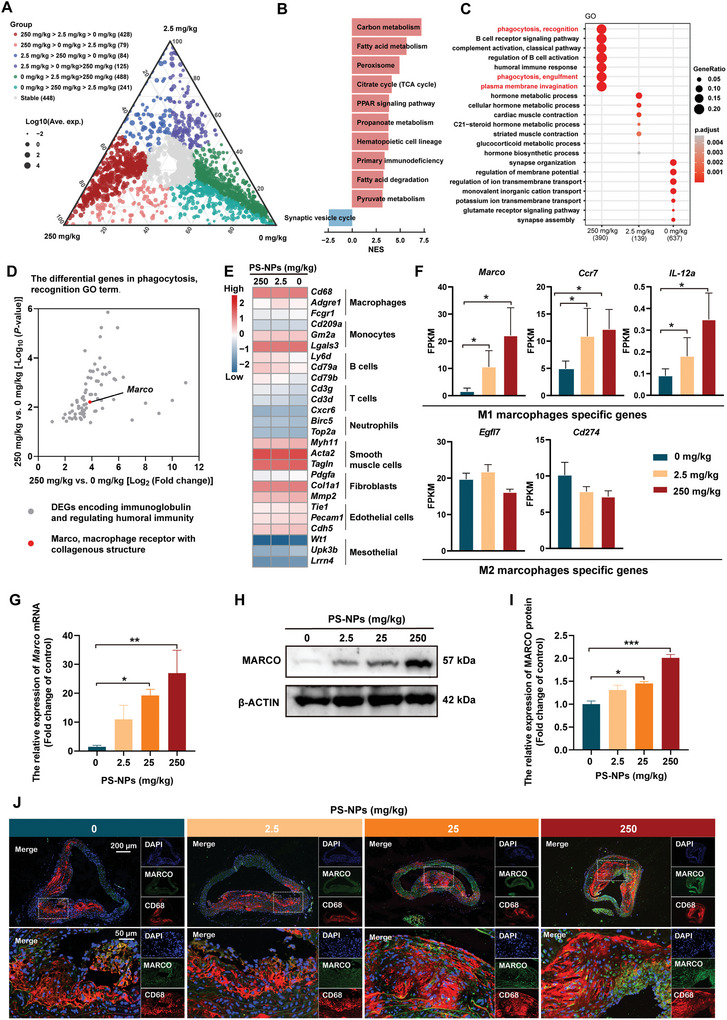
PS‐NPs activated macrophage phagocytosis in the *ApoE* ^−/−^ mice. A) DEGs altered by PS‐NPs in a dose‐dependent manner visualized by the ggtern package in R. B) GSEA of the DEGs (Log_2_FC > 2 and Log_2_FC < −2) highlighted the most activated pathways in carbon metabolism and fatty acid metabolism. C) GO analysis revealed the most affected biological processes of humoral immune response activation and mononuclear macrophage phagocytosis in the 250 mg kg^−1^ group. D) DEGs in the 250 mg kg^−1^ group showed *Marco* increased phagocytosis of macrophages by PS‐NP in the aortas. E) Heatmap showing the highest expressed marker genes of macrophages, monocytes, B cells, T cells, neutrophils, and smooth muscle cells. F) PS‐NPs upregulated specific genes (*Marco*, *Ccr7*, *IL‐12α*) in M1 macrophages and downregulated specific genes (*Egfl7*, *Cd274*) in M2 macrophages. G–I) PS‐NP‐increased expression of Marco mRNA and protein in the mouse aortas. J) IF staining of the aortic arch evidenced by upregulated MARCO overlapped with CD68, a specific macrophage marker. Results are shown as mean ± SD. Comparisons were made with ANOVA, followed by Tukey's method. *P < 0.05, **P < 0.01, and ***P < 0.001, versus the control group. DEGs: differentially expressed genes; GSEA: gene set enrichment analysis; GO: gene ontology; IF: immunofluorescence.

To explore the mechanisms underlying PS‐NP‐caused cardiovascular toxicity in the mice, we performed transcriptomic analysis (**Figure** **3**A–F). The ggtern package in R visualized that most of the differentially expressed genes (DEGs) were in either the 250 mg kg^−1^ > 2.5 mg kg^−1^ > 0 mg kg^−1^ group or the 0 mg kg^−1^ > 2.5 mg kg^−1^ > 250 mg kg^−1^ group (Figure 3A). This result suggested that the majority of the PS‐NP‐altered DEGs trended dose‐dependently. Gene set enrichment analysis (GSEA) highlighted that the carbon metabolism, fatty acid metabolism, and other metabolic pathways were activated (Figure 3B). Gene ontology (GO) analysis revealed that the mostly affected biological processes were the activation of humoral immune response and phagocytosis of the mononuclear macrophage in the 250 mg kg^−1^ group (Figure 3C). Transcriptomic analysis on DEGs in the macrophage phagocytosis‐related pathways revealed that macrophage receptor with collagenous structure (*Marco*) increased phagocytosis of macrophages induced by PS‐NP exposure in the aortas (Figure 3D). Marker gene expressions for different cell types were analyzed with a heatmap, which showed that the highly expressed genes were markers of macrophages, monocytes, B cells, T cells, neutrophils, and smooth muscle cells (Figure 3E). We noted that PS‐NPs upregulated the marker genes of M1 macrophages (*Marco*, *Ccr7*, and *IL‐12α*) and downregulated the marker genes of M2 macrophages (*Egfl7*, *Cd274*) (Figure 3F). These results indicated that PS‐NP exposure had activated the macrophages. Macrophage receptor with collagenous structure (MARCO) mediates the phagocytosis of macrophages.^[^ ^39^ ^]^ Thus, we verified that the PS‐NP increased the expression of Marco mRNA and protein in the aortas (Figure 3G–I). Furthermore, we found that the expression of CD68, a specific maker of macrophages, and MARCO increased in the aortas after PS‐NP exposure (Figure 3J). Taken together, PS‐NP exposure activated macrophages in the mouse aortas.

### 2.6 PS‐NPs Aggravated Lipid Accumulation by Upregulating MARCO In Vitro

**Figure 4:**
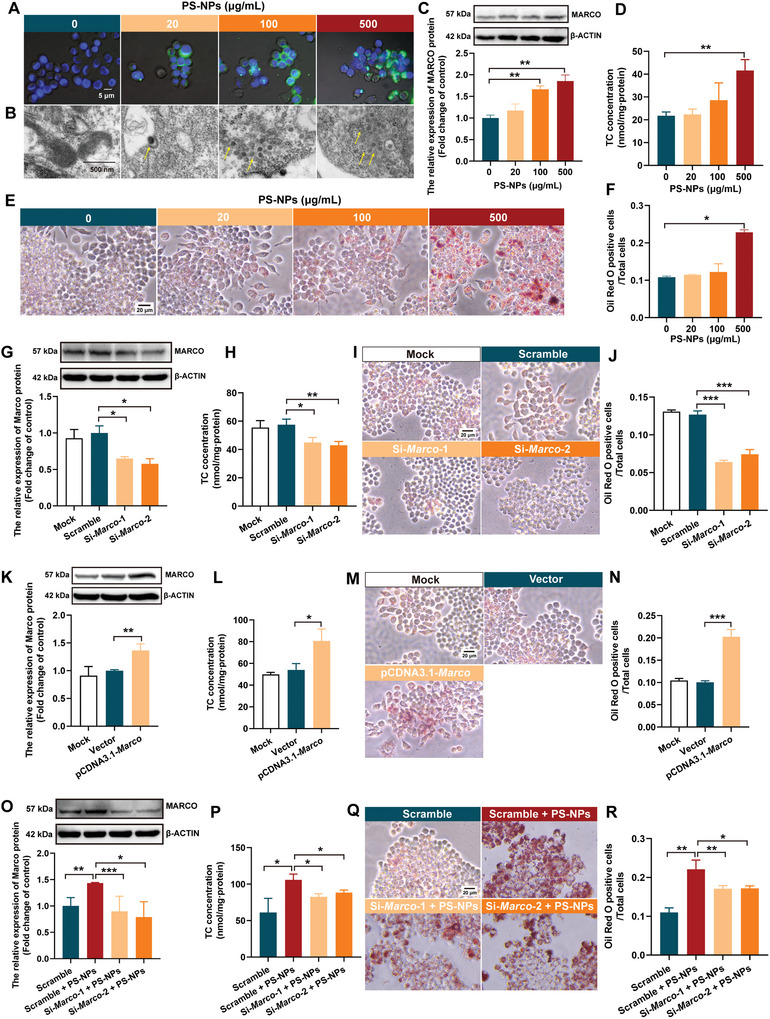
PS‐NPs aggravated lipid accumulation by upregulating MARCO in vitro. A) Green fluorescence PS‐NPs around the nucleus of macrophages after 24 h exposure, and B) increased aggregation of autophagic vacuoles as the PS‐NP concentrations increased (40× magnification). C) PS‐NPs enhanced MARCO expression, D) increased intracellular TC content, and E,F) aggravated intracellular accumulation of lipid droplets under ox‐LDL cotreatment. G) MARCO knockdown resulted in decreases in H) intracellular TC content and I,J) lipid droplets. K) MARCO overexpression caused obvious increases in L) intracellular TC content and M,N) lipid droplets. O–R) Rescue functional experiments showed that MARCO knockdown reversed the PS‐NP‐exacerbated lipid uptake when the macrophages were transfected with si‐*Marco* before treatment with PS‐NPs. Data are expressed as mean ± SD. Comparisons were made with ANOVA, followed by Tukey's method. A Student's *t*‐test was applied to detect the significance of any differences between the two groups. *P < 0.05, **P < 0.01, and ***P < 0.001, versus the control group or the indicated groups. ox‐LDL: oxidized low‐density; TC: total cholesterol.

Macrophage accumulation plays a major role in atherosclerotic plaque progression, which promotes inflammation and aggravates foam cells taking in ox‐LDL.^[^ ^40^ ^]^ To simulate the PS‐NPs’ promotion on atherosclerotic plaque progression in the *ApoE* ^−/−^ mouse model, we used RAW264.7, a murine macrophage cell line, for in vitro mechanistic investigation. In order to observe whether 50 nm PS‐NP particles could match the internalization of macrophages, we used fluorescent PS‐NPs to exam their uptake by macrophages. After exposure for 24 h, we detected green fluorescence around the nuclei of the macrophages (**Figure** **4**A). Furthermore, by using transmission electron microscopy (TEM), we observed PS‐NP particles aggregated in the autophagic vacuoles of cytoplasm, and the particles’ intensity strengthened as the PS‐NP concentrations increased (Figure 4B). To explore the roles of PS‐NPs in the formation of macrophage‐derived foam cells, we applied ox‐LDL to construct a foam cell model. PS‐NPs significantly enhanced the Marco expressions of both mRNA and protein (Figure S3A, Supporting Information; Figure 4C). Lipid uptake assay revealed that PS‐NPs significantly increased intracellular total cholesterol (TC) content (Figure 4D), and also aggravated the intracellular accumulation of lipid droplets under ox‐LDL cotreatment (Figure 4E,F). These in vitro data validated our findings from the mouse study that PS‐NPs upregulated MARCO expression and promoted lipid accumulation.

Next, we explored the role of MARCO in PS‐NP‐aggravated lipid accumulation in macrophage‐derived foam cells by cotreatment with ox‐LDL. We found that MARCO knockdown (Figure S3B, Supporting Information; Figure 4G) resulted in decrease in intracellular TC content (Figure 4H) and lipid droplets (Figure 4I,J). However, the overexpression of MARCO (Figure S3C, Supporting Information; Figure 4K) caused obvious increase in intracellular TC content (Figure 4L) and lipid droplets (Figure 4M,N). In addition, rescue functional experiments showed that MARCO knockdown reversed the PS‐NP‐exacerbated lipid uptake when the foam cells were transfected with si‐*Marco* before treatment with PS‐NPs (Figure S3D, Supporting Information; Figure 4O,R). Collectively, PS‐NPs promoted lipid accumulation by upregulating the MARCO expression in the macrophages in vitro.

### 2.7 PS‐NPs Caused Dyslipidemia in the *ApoE* ^−/−^ Mice

**Figure 5:**
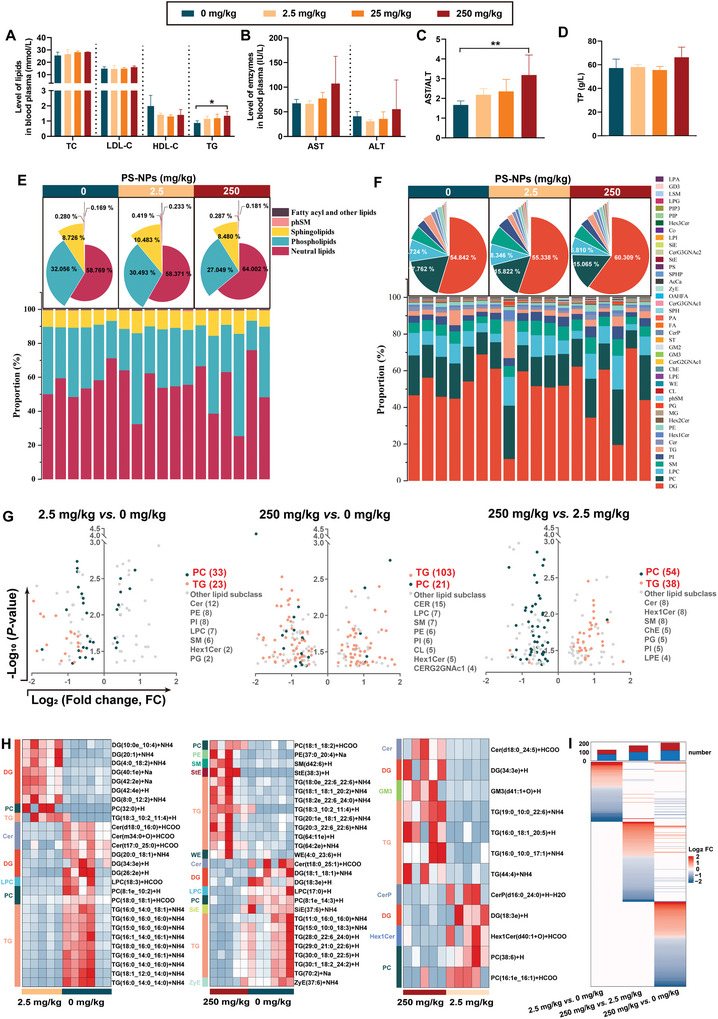
PS‐NPs caused dyslipidemia in the *ApoE* ^−/−^ mice. Mice in the PS‐NP‐exposed groups had elevated A) TG, B) AST, ALT, and C) AST/ALT ratios. D) There was no significant difference in TP levels between the PS‐NP groups and the control. E) At the category level, the major lipid components in plasma were identified as neutral lipids, phospholipids, and sphingolipids, with a decreased proportion of phospholipids and an increased proportion of neutral lipids in the PS‐NP groups. F) At the subclass level, DG, PC, LPC, SM, PI, and TG components had the highest proportion of plasma lipids, with an increased DG proportion and decreased PC and LPC proportions as the PS‐NP dose increased. G) TG and PC were the two subclasses with the most lipid species for all differentially expressed lipid species changed by PS‐NPs. H) The altered lipid species in the 2.5 mg kg^−1^ group were distributed primarily in the DG and TG subclass, while the altered lipid species in the 250 mg kg^−1^ group were distributed primarily in the TG subclass. I) Differentially expressed lipid profiles had little in common in terms of lipid species among the three comparison groups, as shown in the heatmap. Data were expressed as mean ± SD. Comparisons were made with ANOVA, followed by Tukey's method. *P < 0.05 and **P < 0.01, versus the control group. ALT: alanine aminotransferase; AST: aspartate aminotransferase; DG: diglyceride; LPC: lysophosphatidylcholine; PC: phosphatidylcholines; PI: phosphatidylinnositols; SM: sphingomyelin; TG: triglycerides; TP: total protein.

Given the crucial role of blood lipids in the development of macrophage phagocytosis and the progression of atherosclerotic plaque,^[^ ^41^ ^]^ we explored how PS‐NP exposure induces dyslipidemia and therefore contributes to atherosclerosis. The results showed that the mice in the 250 mg kg^−1^ PS‐NP‐exposed group had significantly elevated triglycerides (TG) and aspartate aminotransferase (AST)/alanine aminotransferase (ALT) ratios compared with the control (**Figure** **5**A,C). Although there was no statistical significance, PS‐NPs increased AST and ALT dose‐dependently (Figure 5B). There was no significant difference in the TC, low‐density lipoprotein cholesterol (LDL‐C), high‐density lipoprotein cholesterol (HDL‐C), or total protein (TP) among the control or PS‐NP groups (Figure 5A–D). These biochemistry results indicated that PS‐NP exposure led to dyslipidemia.

### 2.8 PS‐NPs Disrupted Lipid Metabolism in the *ApoE* ^−/−^ Mice

To further explore how the PS‐NPs disrupted lipid metabolism, we used lipidomics to reveal the lipidome change in the mouse plasma due to PS‐NP exposure. The results showed that 3740 lipid species were identified and quantified, covering 5 major categories and 43 subclasses (Table S2, Supporting Information). Orthogonal Partial Least Squares Discriminant Analysis separated groups based on PS‐NP exposure (Figure S4A–C, Supporting Information). There was more variation within groups at the dose of 250 mg kg^−1^ (Figure S4B, Supporting Information), and an outlier in the 2.5 mg kg^−1^ group (Figure S4A, Supporting Information). This outlier also could be found in lipid composition distribution among individuals in the 2.5 mg kg^−1^ group (Figure 5E,F, middle panel). In order to prevent outlier interference, we excluded data from this sample from the rest of the analysis. We also separately analyzed the plasma lipid components at the category and subclass levels. At the category level, the major lipid components in plasma were identified as neutral lipids, phospholipids and sphingolipids (Figure 5E). The proportion of phospholipids decreased with the increasing doses of PS‐NPs. Additionally, the 250 mg kg^−1^ group had a higher neutral lipids proportion than the control (Figure 5E). At the subclass level, diglyceride (DG), PC, LPC, sphingomyelin (SM), phosphatidylinnositols (PI), and TG components with the highest proportions of neutral lipids comprised more than 90% of the lipids (Figure 5F). The DG proportion increased with the increase in PS‐NP doses, but the PC and LPC decreased as the PS‐NP doses increased (Figure 5F).

Then, for all differentially expressed lipid species, the two most abundant subclasses were TG and PC (Figure 5G). Compared with the control, most differential lipid species in the 2.5 mg kg^−1^ group were in the PC subclass, followed by the TG subclass (Figure 5G, left panel); the 250 mg kg^−1^ group had the most lipid species in the TG subclass, followed by the PC subclass (Figure 5G, middle panel). For differential lipid species with large fold changes (FC, Log_2_FC > 1.5 or Log_2_FC < −1.5), we found that the altered lipid species in the 2.5 mg kg^−1^ group was primarily distributed in the DG and the TG subclass, but the altered lipid species in the 250 mg kg^−1^ group was primarily distributed in the TG subclass (Figure 5H). A small number of common differentially expressed lipids responded to PS‐NP exposure in a dose‐dependent manner, primarily in those of TG subclass (Figure S4D, Supporting Information). These results characterized a common feature in which the altered lipid species changed from the other lipid subclass to the TG subclass with the increasing doses of PS‐NPs. In addition, the heatmap showed that the differentially expressed lipid profiles had little in common in terms lipid species in the three comparison groups (Figure 5I). Also, there were more altered lipids in the 250 mg kg^−1^ group than in the 2.5 mg kg^−1^ group, when compared with the control (Figure 5I). Taken together, these results indicated that the PS‐NPs disrupted lipid metabolism in the mice.

### 2.9 Long‐Chain Acyl Carnitines (LCACs) Drove Lipid Accumulation by Upregulating MARCO In Vitro

**Figure 6:**
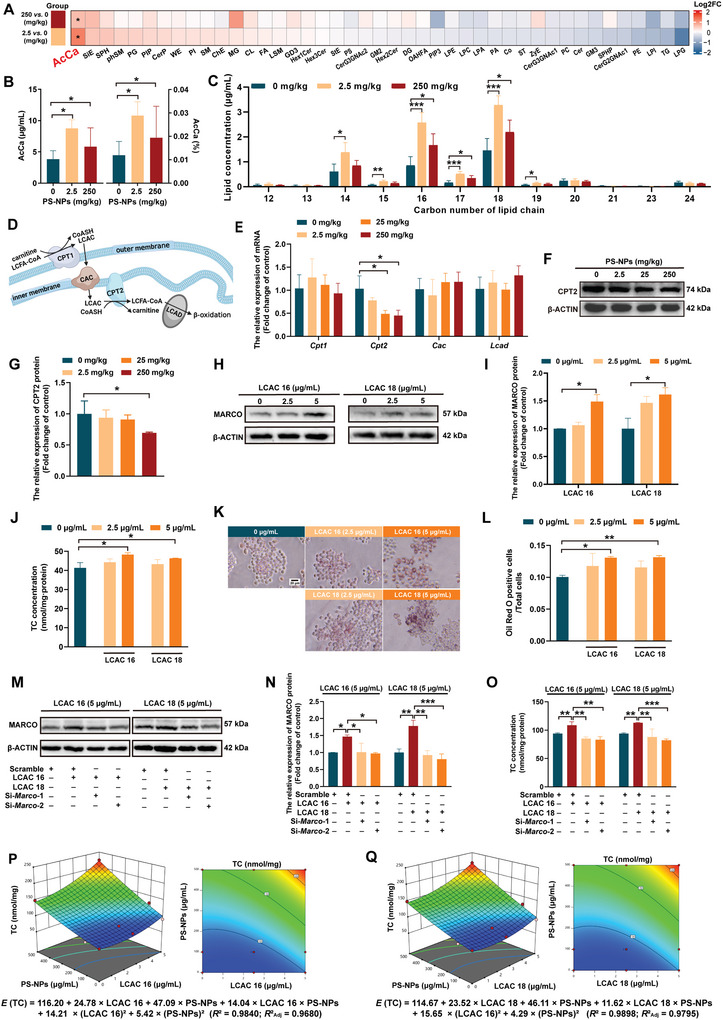
LCACs aggravated lipid accumulation by upregulating MARCO in vitro. A,B) AcCa was the only subclass with a statistical increase in the 2.5 and 250 mg kg^−1^ groups, in terms of both percentage and absolute concentration. C) The most common AcCa chain lengths were 18, 16, and 14 carbons, and they were from the LCACs. D) Fatty acid oxidation disorders can cause substantial increases in LCACs’ plasma concentrations, which are involved with CPT1, CPT2, CAC, and LCAD. E–G) PS‐NPs decreased hepatic CPT2, but not CPT1, CAC or LCAD. H,I) Both of LCAC 16 and LCAC 18 at 5 µg mL^−1^ increased MARCO protein levels. J–L) Increased intracellular TC content and lipid droplets. M–O) MARCO knockdown reversed the LCAC 16‐ and LCAC 18‐enhanced lipid uptake when the foam cells were transfected with si‐MARCO before LCAC 16 and LCAC 18 treatments. P,Q) Synergistic effects on increasing TC in foam cells caused by combination of PS‐NPs and LCACs (LCAC 16 and LCAC 18). Data are expressed as mean ± SD. Comparisons were made with ANOVA, followed by Tukey's method. A Student's *t*‐test was applied to detect any significant difference between the two groups. Synergistic effects of PS‐NPs and LCACs were analyzed by RSM. *P < 0.05, **P < 0.01, and ***P < 0.001, versus the control group or the indicated groups. AcCa: fatty acyl carnitine; CAC: carnitine/acylcarnitine carrier protein; CPT1: carnitine palmitoyltransferase 1; CPT2: carnitine palmitoyltransferase 2; LCACs: long‐chain acyl carnitines; LCAD: long‐chain acyl Co‐A dehydrogenase; RSM: response surface methodology; TC: total cholesterol.

To explore how the PS‐NPs‐disrupted lipid metabolism contributed to atherosclerosis, in the subclass level comparison of plasma lipid components changed by PS‐NP exposure, we identified fatty acyl carnitine (AcCa) as the only subclass with a statistical increase in the 2.5 and 250 mg kg^−1^ groups, in terms of both percentage and absolute concentration, when compared with the control (**Figure** **6**A,B). Moreover, we found that the most common chain lengths among the AcCa were 18, 16, and 14 carbons, that is, the LCACs (Figure 6C). AcCa is intermediated oxidative metabolites that consist of a fatty acid esterified to a carnitine molecule.^[^ ^42^ ^]^ They are generated by both mitochondrial and peroxisomal enzymes, including the carnitine palmitoytransferase 1 (CPT1) and carnitine palmitoyltransferase 2 (CPT2) enzymes, for the purpose of transporting long‐chain fatty acids across the mitochondrial membrane for *β*‐oxidation. Fatty acid oxidation disorders can cause substantial increases in plasma and tissue concentrations of LCACs (Figure 6D).^[^ ^43^ ^]^ CPT2, or long‐chain acyl Co‐A dehydrogenase (LCAD), is a deficient but carnitine/acylcarnitine carrier protein (CAC). CPT1 remains functional in the liver, and is the most common fatty acid oxidation disorder.^[^ ^44^ ^]^ Based on the above information involved in LCACs’ metabolic pathway, we detected the hepatic *Cpt1*, *Cpt2*, *Cac*, and *Lcad* mRNA levels in the mice exposed to PS‐NPs. Interestingly, we found significant decreases in only hepatic CPT2 mRNA and protein (Figure 6E–G), which might be the primary contributor behind the PS‐NP‐activated LCAC accumulation in the mouse plasma.

Elevated LCAC levels have been associated with lipotoxic effects^[^ ^45^ ^]^ and elevated myocardial TG.^[^ ^46^ ^]^ Considering the PS‐NP‐aggravated lipid accumulation via MARCO upregulation both in vitro and in vivo, as well as the association between PS‐NP‐increased LCACs and lipotoxic outcomes, it is reasonable to hypothesize that LCACs aggravate lipid accumulation by upregulating MARCO in the macrophages. To address this notion, we explored the role of LCAC 16 and LCAC 18 (the two LCACs with the highest concentrations) in lipid uptake. Our results showed that both LCAC 16 and LCAC 18 at 5 µg mL^−1^ had increased Marco mRNA and protein levels (Figure 6H,I; Figure S6A, Supporting Information). This finding coincided with an increase in intracellular TC content and lipid droplets (Figure 6J–L). Furthermore, we demonstrated that MARCO knockdown reversed the LCAC 16‐ and LCAC 18‐enhanced lipid uptake when the foam cells were transfected with si‐*Marco* before LCAC 16 and LCAC 18 treatments (Figure 6M–O; Figure S6B, Supporting Information). Collectively, LCACs drove lipid accumulation via upregulating MARCO.

### 2.10 Combination of PS‐NPs and LCACs Synergistically Increased Intracellular TC In Vitro

To investigate the synergistic effects caused by combination of PS‐NPs and LCACs, we employed response surface methodology (RSM) in this study. ANOVA of quadratic models of PS‐NPs and LCACs for mixture design was shown in **Table** **2**. The results indicated that PS‐NPs and the intracellular TC content fit well to a complete quadratic model with LCAC 16 or LCAC 18, respectively. This was evidenced by the *R* ^2^ as 0.9840 and *R* ^2^_Adj_ as 0.9680 for the model with LCAC 16, and *R* ^2^ as 0.9898 and *R* ^2^_Adj_ as 0.9795 for the model with LCAC 18. We found that PS‐NPs exhibited greater effects on increasing the intracellular TC content due to the higher coefficients than those with LCAC 16 (47.09 vs 24.78) or LCAC 18 (46.11 vs 23.52). Interestingly, strong synergistic enhanced effects were observed in combination of PS‐NPs/LCAC 16 or PS‐NPs/LCAC 18, with interaction coefficients as 14.04 and 11.62, respectively (Figure 6P,Q). To sum up, the combination of PS‐NPs and LCACs synergistically increased TC in the ox‐LDL‐activated foam cells.

**Table 2:** ANOVA of quadratic models of LCAC and PS‐NPs for mixture design

Source	Sum of squares	Degree of freedom	Mean square	*F*‐value	*P*‐value
Model (LCAC 16‐PS‐NPs)	21 035.30	5	4207.06	61.52	0.0002^a)^
LCAC 16	3497.34	1	3497.34	51.14	0.0008^a)^
PS‐NPs	13 304.89	1	13 304.89	194.57	<0.0001^a)^
(LCAC 16) × (PS‐NPs)	883.67	1	883.67	12.92	0.0156^a)^
(LCAC 16)^2^	511.33	1	511.33	7.48	0.0411^a)^
(PS‐NPs) ^2^	26.41	1	26.41	0.3863	0.5615
Residual error	4.36	2	2.18	–	–
Total	21 377.21	10	–	–	–
	*R* ^2^ = 0.9840; *R* ^2^_Adj_ = 0.9680				
Model (LCAC 18‐PS‐NPs)	19 943.90	5	3988.78	96.56	<0.0001^a)^
LCAC 18	3150.86	1	3150.86	76.28	0.0003^a)^
PS‐NPs	12 759.23	1	12 759.23	308.89	<0.0001^a)^
(LCAC 18) × (PS‐NPs)	605.10	1	605.10	14.65	0.0123^a)^
(LCAC 18)^2^	620.84	1	620.84	15.03	0.0117^a)^
(PS‐NPs) ^2^	16.57	1	16.57	0.4011	0.5543
Residual error	1.11	2	0.56	–	–
Total	20 150.43	10	–	–	–
	*R* ^2^ = 0.9898; *R* ^2^_Adj_ = 0.9795				

^a)^ Significant (*P* < 0.05); –: Not applicable; Adj: adjusted; LCAC: long‐chain acyl carnitine.

## 3 Discussion

**Scheme 1:**
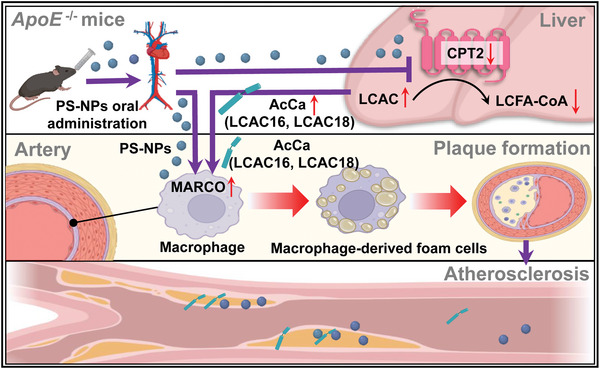
Schematic diagram of the present study. This study indicated that LCACs (LCAC 16 and LCAC 18) aggravated PS‐NP‐induced plaque formation in the aorta by upregulating MARCO, leading to atherosclerosis. Increased lipid uptake by PS‐NP and LCAC exposure in vitro. Increased TC levels in the foam cells when they were coexposed to PS‐NPs and LCACs.

We have documented MNP‐induced atherosclerotic vascular injury,^[^ ^47^ ^]^ but the mechanisms remain unclear. We performed both transcriptomics and lipidomics analyses to provide comprehensive insight into PS‐NP‐driven atherosclerotic vascular injury in the HFD‐fed *ApoE* ^−/−^ mice. Our findings showed that MARCO upregulation, macrophage activation, and lipid metabolism disruption were the lynchpin in the PS‐NP‐induced atherosclerotic vascular damage in vivo. Furthermore, we also found that LCACs aggravated PS‐NP‐induced atherosclerotic vascular injury by upregulating MARCO and macrophage activation in vitro. Thus, our study represents a crucial step toward understanding how NPs damage the aorta (**Scheme** **1**), and also highlights the combined effects of MNPs with endogenous metabolites on the cardiovascular system.

MARCO has been linked to atherosclerosis, both experimentally and epidemiologically.^[^ ^48^, ^49^ ^]^ As a member of the class A scavenger receptor family, MARCO can bind to the modified LDL and VLDL.^[^ ^50^, ^51^, ^52^ ^]^ Thus, MARCO promotes lipoprotein and cholesterol deposition in artery walls, and promotes the formation of atherosclerotic plaque.^[^ ^39^, ^53^ ^]^ Also, as a well‐known M1 macrophage marker, MARCO upregulation activates phagocytosis and increases lipid uptake in macrophages,^[^ ^54^ ^]^ which converts macrophages into foam cells. The foam cells subsequently contribute to plaque instability and rupture.^[^ ^55^ ^]^ This is the crucial determinant of the initiation and progression of atherosclerotic lesions.^[^ ^56^ ^]^ Our transcriptomic data highlighted MARCO upregulation after PS‐NP exposure, which contributed to the atherosclerosis formation. Indeed, MARCO mediates the recognition and ingestion of environmental particulate matters including PS‐NPs.^[^ ^57^, ^58^ ^]^ As expected, the present study revealed MARCO upregulation in the aorta after PS‐NP exposure. Thus, MARCO plays a crucial role in PS‐NP‐induced atherosclerosis.

Lipids are one of the most important stimuli initiating atherogenesis.^[^ ^59^ ^]^ PS‐NP‐altered lipid metabolism has been found in both marine invertebrates and fish.^[^ ^60^, ^61^, ^62^ ^]^ However, whether NPs cause lipid metabolism disorder, and how lipid metabolism disorder is involved in the progression of atherosclerosis in mammals remains unclear. In this study, we found that the PS‐NP‐induced lipid metabolism alteration may be involved in the progression of atherosclerosis. First, PS‐NPs provoked a higher neutral lipid proportion in the blood. This may increase the risk of neutral lipids being trapped and retained within the intima, which is involved in atherosclerotic lesion development.^[^ ^63^ ^]^ Among the disturbed neutral lipids induced by PS‐NPs, TG constituted the majority. TG‐rich lipoproteins can be captured by the scavenger receptors of macrophages in the arterial wall without oxidative modification, while other lipoproteins cannot.^[^ ^64^, ^65^, ^66^ ^]^ This makes TG a strong and independent predictor of atherosclerotic cardiovascular disease.^[^ ^67^, ^68^ ^]^ Our results indicated that PS‐NPs may aggravate atherosclerosis by disturbing the metabolism of neutral lipids, particularly TG. Second, we found that PS‐NPs decreased the proportion of phospholipids in the blood. Phospholipid‐based HDLs play a protective role in atherosclerosis, where they can improve cholesterol efflux capacity, reduce cholesterol crystals, and inhibit inflammation in the arterial wall.^[^ ^69^, ^70^ ^]^ Therefore, the low phospholipid level induced by PS‐NPs may also be one of the reasons that PS‐NPs promote atherosclerosis progression.

LCACs are the intermediate oxidative metabolites of long‐chain fatty acid (>14 carbon atoms). Fatty acid oxidation disorders lead to LCAC accumulation.^[^ ^71^, ^72^, ^73^ ^]^ Moreover, excessive LCACs has been shown to be lipotoxic, leading to lipid accumulation in the myocardium^[^ ^46^ ^]^ and increased interleukin 6 in myotube cells.^[^ ^74^ ^]^ Furthermore, fatty acylcarnitines, including LCACs, play a key metabolic role in initiating an immune response in mononuclear phagocytes.^[^ ^75^ ^]^ LCACs can promote M1 macrophage polarization proinflammatory cytokine secretion by activating NF‐*κ*B and inflammation‐associated pathways,^[^ ^76^ ^]^ and by inhibiting AMPK pathways.^[^ ^77^ ^]^ Similar to the PS‐NPs, we found that LCACs targeted MARCO and then promoted the phagocytosis on lipids in RAW264.7 cells. This may partially be attributed to LCACs’ proinflammatory effect. Interestingly, we observed that the combination of PS‐NPs and LCACs triggered more lipid uptake than each on its own in RAW264.7 cells. This synergistic effect is conceptually similar to the finding that the combination of PS‐NPs and ketoconazole (KCZ)/fluconazole (FCZ) induced larger declines in the heart rate of zebrafish embryos, for all exposed groups than for KCZ/FCZ alone or in combination.^[^ ^78^ ^]^ It is also conceptually similar to the finding that the combination of PS‐NPs and chloroauric acid hydrated (HAuCl_4_) causes more severe underdeveloped hearts and more severe yolk edema in zebrafish embryos than those with PS‐NP exposure alone.^[^ ^79^ ^]^ Thus, our findings will stimulate more interest in exploring whether these combined effects of NPs are cooperative, not only with xenobiotic compounds, but also with endogenous metabolites.

In the present study, the mice were exposed to PS‐NPs through oral gavage because human exposure to MNPs largely occurs through ingestion.^[^ ^80^ ^]^ The doses of 2.5 and 25 mg kg^−1^ PS‐NPs were applied to the mice, based on 10 mg kg^−1^ BW day^−1^ for human ingestion.^[^ ^7^, ^8^, ^81^ ^]^ Moreover, we set a higher exposure level of 250 mg kg^−1^ PS‐NPs, considering the unrelenting rise in MNP exposure levels in the environment.^[^ ^82^, ^83^ ^]^ The concentration of MNPs in human blood has been reported to be about 1.6 µg mL^−1^.^[^ ^3^ ^]^ The mouse blood PS‐NP levels of the three exposed groups were 0.17, 0.51, and 3.37 µg mL^−1^, which confirmed that the present PS‐NP exposure levels by oral administration in the mice were relevant to human exposure levels. Based on this human data and taking into account uncertainties such as exposure duration and others, we set the low dose of 20 µg mL^−1^ to be closed ten times the MNP concentration in the human blood. We also set two high‐dose groups of 100, and 500 µg mL^−1^ with a fivefold difference for dose‐dependent hazard identification.

Some limitations in the present study should be noted. First, we used a sensitive mouse model of HFD‐fed *ApoE* ^−/−^ mice to simulate the high‐risk atherosclerosis population. Caution should be taken when extrapolating our findings to the general population. Second, we used PS‐NPs in the present study, as PS is one of the most abundant and typical MNPs.^[^ ^84^, ^85^ ^]^ It is worth noting that the particle material is a major factor affecting the MNP toxicity, because various plastic types in realistic environments likely cause different toxic effects by MNPs.^[^ ^86^ ^]^ We also used 50 nm particles as the representative NPs. Particle size is another major factor which influences MNP toxicity.^[^ ^87^ ^]^ Moreover, although the PS‐NP exposure level in the mouse blood was similar to that of human, lower environmentally realistic doses and long‐term MNP exposure should be encouraged. Therefore, more work is needed to clarify the cardiovascular toxicities of MNPs of different sizes, materials, and durations.^[^ ^47^ ^]^ Furthermore, we initially investigated the PS‐NP levels in the mouse blood by applying single fluorescence PS‐NP exposure. More dynamic exposures are needed to investigate MNPs’ ADME (absorption, distribution, metabolism, and excretion). Although we found PS‐NP‐induced artery stiffness using UBM, few DEGs were related to artery stiffness in our transcriptomic analysis. Thus, the mechanisms underlying PS‐NP‐exacerbated vascular stiffness in the *ApoE* ^−/−^ mice are worth for further investigation. Finally, given the numerous lipids which may be involved in the PS‐NP‐induced atherosclerosis, future studies are needed to explore the unknown roles and modes of action of many individual lipids, and their combinations, that may facilitate PS‐NP‐induced atherosclerosis. In addition to the combined cardiovascular toxicity of MNPs and other pollutants,^[^ ^78^, ^79^ ^]^ the combined effects of MNPs with endogenous metabolites on the cardiovascular system also warrant further study.

In conclusion, this study revealed that LCACs aggravated PS‐NP‐induced atherosclerosis by upregulating MARCO in *ApoE* ^−/−^ mice fed with HFD. The results of this study offer new insights into the mechanisms underlying MNP‐induced cardiovascular toxicity, and will help in managing the growing cardiovascular risks of MNP exposure in humans.

## 4 Experimental Section

### Nanoplastics and Chemicals

50 nm pristine nonfluorescence PS‐NP and yellow‐green fluorescence PS‐NP suspensions were purchased from Magsphere (Pasadena, CA, USA). The stock solution contained 10% suspensions in deionized water. The density of PS‐NPs was 1.05 g mL^−1^ based on the product's Certificate of Analysis. The characteristics of both PS‐NP particles have been described previously.^[^ ^7^ ^]^ Other chemicals were purchased from commercial sources with the highest available purity.

### Ethical Approval

All animal experimentation was performed following the National Guidelines for Animal Care and Use of China. The Southern Medical University Scientific Research Committee on Ethics in the Care and Use of Laboratory animals has approved this study (Permit No. SMUL2021164).

### Animals and Experimental Design

*ApoE* ^−/−^ mice at the age of 1–2 months are commonly used as animal models for studying spontaneous atherosclerosis.^[^ ^88^ ^]^ A total of 130 male *ApoE* ^−/−^ mice (age, eight‐week; weight, 18–22 g) were obtained from the Guangdong Medical Laboratory Animal Center (Guangzhou, China) to assess the long‐term effects of PS‐NP exposure on atherosclerosis. All animals were housed and acclimatized in sterilized filter‐topped cages with food and water accessed ad libitum. They were maintained in a specific pathogen‐free facility with constant humidity (50 ± 5%) and temperature (24 ± 1 °C), at a 12/12 h light/dark cycle for 1 week. Study design and the number of mice for each experimental group are listed in **Table** **3**. In Experiment 1, to characterize the PS‐NP particles in foods, 6 mice were randomly assigned into two groups (*n* = 3 in the control and the 250 mg kg^−1^ group); to monitor the exposure levels of various PS‐NP concentrations, 24 mice were randomly assigned into four groups (*n* = 6 per group). They were treated with a single oral gavage with either double distilled water as a control, or with fluorescence PS‐NPs at doses of 2.5, 25, or 250 mg kg^−1^, at a volume of 20 mL kg^−1^ BW after fasting for 12 h. At 24 h postgavage, the mice with 3% pentobarbital were anesthetized, and the blood from the retroorbital plexus was collected in an ethylene diamine tetraacetic acid anticoagulation tube, and then plasma from the blood was separated and it was stored at −80 °C until use. After perfusion with saline, aorta and liver tissues were collected on ice. The blood and histological aorta and liver slides were both used for fluorescence PS‐NP detection.

**Table 3:** Experimental design for *ApoE* ^−/−^ mice

Experimental group	1^a)^				2^b)^				3^b)^			
PS‐NP dose [mg kg^−1^]	0	2.5	25	250	0	2.5	25	250	0	2.5	25	250
Total number of mice (*n*)	9	6	6	9	10	10	10	10	15	15	15	15
Particle characterization and distribution (*n*)^c)^	9	6	6	9	–^l)^	–	–	–	–	–	–	–
Blood pressure (*n*)^d)^	–	–	–	–	5	5	5	5	–	–	–	–
UBM (*n*)^e)^	–	–	–	–	10	10	10	10	–	–	–	–
Gross pathology (*n*)^f)^	–	–	–	–	5	5	5	5	–	–	–	–
Section staining and IF (*n*)^g)^	–	–	–	–	5	5	5	5	–	–	–	–
Plasma lipidomics (*n*)^h)^	–	–	–	–	6	6	6	6	–	–	–	–
Aorta WB (*n*)^i)^	–	–	–	–	–	–	–	–	5	5	5	5
Aorta RNA‐seq and qPCR (*n*)^j)^	–	–	–	–	–	–	–	–	10	10	10	10
Liver qPCR and WB (*n*)^k)^	–	–	–	–	–	–	–	–	10	10	10	10

^a)^ Experiment 1: Mice were exposed to PS‐NPs for 30 min for particle characterization in foods, and for 24 h for particle distribution;

^b)^ Experiments 2 and 3: Mice fed with HFD were exposed to PS‐NPs for 19 weeks;

^c)^ Results in Figure 2A,B; Figures S1 and S5 and Table S1 of the Supporting Information;

^d)^ Results in Figure 2C,D;

^e)^ Results in Figure 2E–H;

^f)^ Results in Figure 2I,J;

^g)^ Results in HE staining (Figure 2K,L), Masson staining (Figure 2M,N), Oil Red O staining (Figure 2O,P), and IF (Figure 3I);

^h)^ Results in Figures 5A–I and 6A–C; Figure S3A–D of the Supporting Information;

^i)^ Results in Figure 3H,I;

^j)^ Results in Figure 3A–G;

^k)^ Results in Figure 6E–G;

^l)^ Not available. IF: immunofluorescence; qPCR: quantitative polymerase chain reaction; UBM: ultrasound biomicroscope; WB: western blot.

In Experiments 2 and 3, 100 mice were randomly assigned into two batches of four groups (*n* = 10 per group in Experiment 2, and *n* = 15 per group in Experiment 3), including a control group and three nonfluorescence PS‐NP groups with dosages of 2.5, 25, or 250 mg kg^−1^ in each batch (Table 3). The mice in the exposure groups were administered with nonfluorescence PS‐NP suspension daily by gavage for 19 consecutive weeks; and the mice in the control group were treated with the same volume of distilled water. During the experiments, all mice were fed with HFD (Western diet, 21% fat, g/g, Guangdong Medical Laboratory Animal Center), and received drinking water ad libitum. The mice were monitored and weighed weekly (Table S3, Supporting Information). At the termination of the experiment, the mice were fasted overnight, and the mice were anesthetized with 3% pentobarbital to collect aortas and livers. Blood was collected, as mentioned above, to use for lipid profiles and lipidome analysis.

### PS‐NP Particle Characterization

The size distribution and zeta‐potential of PS‐NP suspensions were detected by a dynamic light scattering with a Zetasizer Nano ZS (Malvern Panalytical GmbH, Kassel, Germany). PS‐NPs were suspended in distilled water and simulated gastrointestinal digestion was performed with the in vitro digestive tract model.^[^ ^89^ ^]^ The digestive fluids and food matrix were prepared by following previously described protocols.^[^ ^89^, ^90^ ^]^ In the context of human MNP intake (5 g week^−1^)^[^ ^81^ ^]^ and fluid intake (2 L day^−1^),^[^ ^91^ ^]^ 500 µg mL^−1^ PS‐NPs in the in vitro study was chosen. This dose was similar to the MNP doses applied in other studies with in vitro digestive tract model.^[^ ^92^, ^93^, ^94^ ^]^ For particle characterization in vivo, we treated the *ApoE* ^−/−^ mice (*n* = 3 per group) with a single oral gavage with either double distilled water or 250 mg kg^−1^ PS‐NP suspension. Based on previous studies,^[^ ^95^, ^96^ ^]^ the mice were anesthetized with 3% pentobarbital at 30 min postexposure, and the blood and stomach contents were collected from the mice for PS‐NP particle characterization.

### Blood Pressure Measurement

Blood pressure was measured (*n* = 5 per group for each test) three times at the 2‐week, 10‐week, and 19‐week, time‐points. Atherosclerosis causes loss of vasomotor activity, disproportionate vascular contractility, and elevated blood pressure.^[^ ^97^ ^]^ Therefore, noninvasive tail‐cuff (Medlab, Calvin Biotechnology, Nanjing, China) was adopted to record blood pressure.^[^ ^98^ ^]^ One week before the experiment, the mice were trained to adapt to the procedures and environment of daily blood pressure measurement. When the emotional state of the mice was stable, the SBP and DBP of each mouse were continually measured, each time with three readings.

### Ultrasound Biomicroscopy

UBM (*n* = 10 per group) was performed one day before terminating the experiment. To assess the stiffness of carotid artery, an ultrahigh‐resolution color doppler Vevo 2100 ultrasound system (FUJIFILM Visualsonics, Washington, USA) equipped with MS 400 mechanical transducers was used to measure the ultrasound imaging parameters of the LCCA. Before the measurement, any hair from the anterior chest wall was carefully shaved. The mice were anesthetized with inhaled isoflurane gas resulting in a heart rate of ≈500 beats min^−1^. The mean diameter and IMT were measured with the Vevo LAB (Fujifilm Visualsonics). IMT was measured with the vascular lumen‐intimal interface selected as the internal measurement site and the media adventitial interface as the external limit. Furthermore, global radial strain rate and global longitudinal strain rate were measured by analyzing the electrocardiography kilohertz‐based visualization 2D dynamic image using advanced speckle tracking algorithms. The identity of mouse was blinded to the operator who analyzed the images, and the measurements were repeated three times.

### Lipid Profile Analysis and Liver Function Assessment

Plasma was obtained by centrifuging blood samples (*n* = 6 per group) at 3000 × *g*, 4 °C for 10 min. TC, TG, HDL‐C, LDL‐C, AST, ALT, and TP were measured, using a multifunctional benchtop clinical chemistry analyzer (Mindray, Shenzhen, China).

### PS‐NP Fluorescence Detection in the Blood, Liver, and Aortic Slides

Fluorescence PS‐NP particles in the blood, liver, and aortic slides were detected in mice (*n* = 6 per group) using the detailed protocol described in the previous study.^[^ ^7^ ^]^ The blood was diluted with 9 mL double distilled water per gram of blood. Serial dilutions of fluorescent PS‐NP particles in the blood were prepared and measured by a fluorescence spectrophotometer (Tecan Spark, Austria) with excitation = 480 nm and emission = 525 nm. The standard curve was established using fluorescent PS‐NP particles in the mouse blood of the control group (Figure S1, Supporting Information). This standard curve was used for calculating PS‐NP concentrations in the mouse blood of the exposed groups.

The liver and aortic tissues were frozen overnight and infiltrated with a series of 10%, 20%, and 30% sucrose solution (Sigma‐Aldrich, Taufkirchen, Germany). Then, the tissues were embedded in optimal cutting temperature (OCT) compound (Tissue‐Tek, Sakura Finetek Japan Co., Ltd., Tokyo, Japan). The cross‐sections were used for fluorescence detection. Slides were scanned and the images were reviewed with the Pannoramic MIDI (3D HISTECH, Budapest, Hungary).

### Histopathological Examination and Immunofluorescence (IF) Staining

To detect the lesion area of the entire aorta, Oil Red O staining was performed on the whole aorta (*n* = 5 per group). After detaching the entire aorta and removing any excess adipose tissue, it was cut longitudinally and was stained with Oil Red O. Then the aorta was placed in 75% alcohol to clean the artery wall without lesions. To detect the pathological component of any lesions in the aortic arch, the aortic arches were immersed in 4% paraformaldehyde (PFA) for 24 h and embedded into paraffin or OCT for histological examination. The cross‐sections of the aortic arches were stained with HE, Oil Red O, and Masson dye. Frozen sections were used for Oil Red O staining, and paraffin sections were used for HE and Masson staining. IF double‐staining was conducted using the same paraffin‐embedded specimens from the aortic arches (*n* = 5 per group) as in the above HE staining. After deparaffinizing and rehydrating the paraffin sections, proteinase K (Servicebio) was used for antigen retrieval, and then 3% hydrogen peroxide (Sinopharm, Beijing, China) and 3% bovine serum albumin (G5001, Servicebio) were used to block the slides for 30 min in sequence. The slides were incubated overnight with primary antibodies of anti‐CD68 (Immunoway, Plano, TX, USA) and anti‐MARCO (Abcam, Cambridge, UK). Next, they were washed three times with phosphate buffered solution (PBS) (pH = 7.4), and then incubated with fluorescent secondary antibodies. The primary and secondary antibodies were listed in Table S4 of the Supporting Information. After nuclear staining with 4′,6‐diamidino‐2‐phenylin‐dole (DAPI) solution (G1012, Servicebio), the slides were mounted with coverslips and scanned by the Pannoramic MIDI and the images were reviewed with Pannoramic Viewer. Finally, the number of CD68 positive cells was calculated and it was normalized to the DAPI count. The MARCO protein level was measured by fluorescent intensity normalized to the DAPI intensity.

### Bulk RNA‐seq

Total RNA was extracted with Trizol (Invitrogen, CA, USA) from mouse aortas (*n* = 10 per group) using a high‐speed low temperature tissue homogenizer (Servicebio) at 60 Hz for 3 min at 4 °C. The RNA integrity number was inspected for RNA integrity using an Agilent 2100 Bioanalyzer (Agilent technologies, Santa Clara, CA, US). Total RNA was further purified and qualified with an RNAClean XP Kit (Beckman Coulter, CA, USA) and RNase‐Free DNase Set (QIAGEN, GmBH, Germany).

Strand‐specific libraries were prepared with a VAHTS Universal V6 RNA‐seq Library Preparation Kit for Illumina (Vazyme, Nanjing, China) so that strand‐specific libraries could be prepared according to the manufacturer's instructions. Sequencing read counts were calculated with Stringtie (v.1.3.0).^[^ ^99^ ^]^ Then the expression level was normalized from different samples using the trimmed mean of M values method,^[^ ^100^ ^]^ and converted the normalized expression levels of samples into fragments per kilobase of transcript per million mapped fragments. The difference of gene expression between groups was analyzed, the *P*‐values were calculated, and multiple hypothesis tests were performed with the edgeR^[^ ^101^ ^]^ (v.3.32.1) package of R. GO enrichment analysis (http://www.geneontology.org/) was performed with false discovery rate ≤0.05 as a threshold.

### Lipidomic Sample Preparation and Lipidome Analysis

Lipids were extracted from mice in each group (*n* = 10 per group) according to the methyl tertiary butyl ethers (MTBE) method.^[^ ^102^ ^]^ First, 50 µL of plasma samples with 200 µL distill water and 20 µL lipid isotope internal standards (Avanti POLAR LIPIDS, INC., AL, USA) were spiked (Table S5, Supporting Information). After adding 240 µL methanol (Thermo Fisher Scientific, Waltham, USA) to the samples, 800 µL of MTBE was added to the mixture and an ultrasound was taken for 20 min at 4 °C followed by another sitting still for 30 min at room temperature. The solution was centrifuged at 14 000 × *g* for 15 min at 10 °C to provide the upper organic solvent layer, and then it was dried under nitrogen.

Reverse phase chromatography was selected for liquid chromatography separation using a CSH C18 column (1.7 µm, 2.1 mm × 100 mm, Waters, Milford, USA). Then, the lipid extracts were redissolved in 200 µL 90% isopropanol/acetonitrile (Thermo Fisher Scientific), and centrifuged at 14 000 × *g* for 15 min. Finally, they received a 3 µL injection of the sample. Solvent A was acetonitrile–water (6:4, v/v) with 0.1% formic acid (Sigma, Saint Louis, USA) and 0.1 mm ammonium formate (Sigma), and solvent B was acetonitrile–isopropanol (1:9, v/v) with 0.1% formic acid and 0.1 mm ammonium formate (Sigma). The initial mobile phase was 30% solvent B at a flow rate of 300 µL min^−1^. It lasted for 2 min, and then linearly increased to 100% solvent B in 23 min, followed by equilibrating at 5% solvent B for 10 min. Mass spectra were acquired by Q‐Exactive Plus in the positive and negative modes. Preset electrospray ionization parameters for all measurements were optimized as follows: source temperature: 300 °C; capillary temperature: 350 °C; ion spray voltage: 3000 V; S‐Lens RF Level: 50%; and the instruments’ scan rage was m/z 200–1800. Finally, the raw data were processed by LipidSearch (Thermo Fisher Scientific) for lipid component identification and alignment.

### Cell Culture and PS‐NP Exposure

The mouse macrophage cell line RAW264.7 was purchased from the American Type Culture Collection, and Mycoplasma contamination test was performed using a One‐step Quickcolor Mycoplasma Detection Kit (Yise Medical Technology, Shanghai, China) every 3 months. The cells were cultured in Dulbecco's modified Eagle's medium (Gibco, CA, USA) and supplemented with 10% (v/v) fetal bovine serum (Gibco) and 100 mg mL^−1^ streptomycin, plus 100 UI mL^−1^ of penicillin (Gibco) in a humidified atmosphere with 5% CO_2_ at 37 °C. To assay the lipid uptake, the cells were seeded on six‐well plates at a density of 7 × 10^5^ cells, and treated with 100 µg mL^−1^ ox‐LDL (Yiyuan, Guangzhou, China)^[^ ^103^ ^]^ combined with PS‐NPs at concentrations of either 20, 100 or 500 µg mL^−1^ for 48 h. The cells were also treated with 100 µg mL^−1^ ox‐LDL and LCACs (Avanti) at concentrations of either 2.5 or 5 µg mL^−1^ for 48 h to measure the lipid uptake capacity. When coexposing the cells to PS‐NPs and LCACs to assay lipid uptake, LCAC 16 and LCAC 18 were applied at a dose of 5 µg mL^−1^, and PS‐NPs at a dose of 500 µg mL^−1^. Some received either the LCAC or the PS‐NPs, while others received both. The LCAC doses (2.5 and 5 µg mL^−1^) applied in the cells were similar to the LCAC levels in the mouse blood detected in the present study (Figure 6C). The PS‐NP treatment doses (20, 100, and 500 µg mL^−1^) used in the cells, and the termination of LCAC and PS‐NP exposures at 48 h of post‐treatment.

### PS‐NP Internalization Assessment

The cells were seeded on coverslips at a density of 1 × 10^5^ cells, and incubated overnight (*n* = 3 per group). They were treated with yellow‐green fluorescent PS‐NPs at concentrations of 0, 20, 100 or 500 µg mL^−1^ for 48 h. Then, the cells were washed twice with PBS and added DAPI (Solarbio, Beijing, China) solution onto the coverslips, followed by observing them under a fluorescence microscope (Nikon Eclipse C1, Tokyo, Japan).

### TEM

To observe PS‐NPs in the intracellular microstructure, TEM was used to assess the morphology of RAW264.7 cells (*n* = 3 per group). The cell precipitation was collected after centrifugation at 1500 × *g* for 5 min, and then a TEM fixative was added and the precipitation was resuspended in the fixative. After agarose pre‐embedding, postfixing, polymerization, ultrathin sectioning, and staining, the cells were observed, and TEM images were taken with a Hitachi HT7700 transmission electron microscope (Hitachi Ltd., Tokyo, Japan).

### Intracellular Lipid Measurement

The TC intracellular content was measured by a total cholesterol assay kit (Applygen, Beijing, China), and the intracellular TC content was calibrated using protein mass. The proteins extracted from the cells were quantified with a Bradford Protein Assay Kit (Bio‐Rad Laboratories, CA, USA). Additionally, intracellular lipid droplets were determined by Oil Red O staining using an Oil Red O stain kit for cultured cells (Solarbio). After washing it with PBS twice, the RAW264.7 cells were fixed with 4% PFA for 20 min and washed for 5 min with 60% isopropanol. Then, they were stained with Oil Red O working solution for 20 min. Finally, any excess dye was washed away with distilled water five times, and the cells were observed under a microscope (Nikon Eclipse C1).

### RNA Interference and Overexpression

Small interfering RNA (siRNA) and overexpression plasmid of *Marco* designed and synthesized by Tsingke Biological Technology (Beijing, China) (Table S6, Supporting Information) were applied to explore MARCO's role in the PS‐NP‐induced increase in lipid uptake. To construct a plasmid overexpressing MARCO, the full‐length mouse MARCO gene cDNA was digested with NotI and XhoI (Thermo Fisher Scientific), and then cloned into pCDNA3.1(+) to generate MARCO overexpression plasmid. Two independent siRNAs targeting the *Marco* gene were purchased from Tsingke. All constructions were verified by sequence analysis. The RAW264.7 cells were plated onto 6‐well plates at ≈50–70% confluence 24 h before transfection. Then, RNA transfection was performed with RNAFit (HanBio, Shanghai, China). After that, the siRNA was diluted to 100 nm. Plasmid transfection was performed with 1.6 µg mL^−1^ LipoFit 3.0 (HanBio).

### Quantitative Polymerase Chain Reaction (qPCR) Analysis

The extracted total RNA from mouse aortas and livers (*n* = 10 per group) in bulk RNA‐seq was also used for qPCR. mRNA was reversely transcribed to cDNA using an Evo M‐MLV RT Master Mix (Accurate Biotechnology, Hunan, China). qPCR was performed using a 5× Priescript RT Master Mix and an SYBR Green Realtime PCR Master Mix (Takara, Shiga, Japan) with an Applied Biosystems ViiA 7 Real‐Time PCR (Thermo Fisher Scientific). The 2^−ΔΔCT^ method^[^ ^104^ ^]^ was adopted to calculate the tested genes which were normalized by *β*‐actin. The sequence of mRNA primers (synthesized by Tsingke) used in this study was presented in Table S7 of the Supporting Information.

### Western Blot

For animal protein, frozen aortic tissues and liver tissues (*n* = 5 per group) were ground with ice‐cold RIPA buffer (Beyotime, Shanghai, China) containing 1:100 protease and phosphatase inhibitor cocktail (Keygen, Nanjing, China) using a high‐speed low temperature tissue homogenizer at 60 Hz for 3 min at 4 °C. Then they were incubated on ice for 30 min. For cell culture protein, the cells were washed with precooled PBS and collected (*n* = 3 per group). Next, they were added into ice‐cold RIPA buffer and incubated on ice for 30 min, as above. After incubation, either for animal protein or for cellular protein, the homogenates were centrifuged at 13 000 × *g* for 30 min at 4 °C, and the supernatant was collected. The supernatant's protein concentration was quantified with a Bradford Protein Assay Kit. Then, the proteins were electrophoresed in 12% SDS‐PAGE and transferred into a polyvinylidene fluoride membrane (Bio‐Rad). After blocking with 5% (w/v) skim milk in Tris buffered saline tween for 2 h at room temperature, the membranes were separately incubated with primary antibodies (anti‐GAPDH, anti‐MARCO and anti‐CTP2) at 4 °C overnight, and then incubated with the appropriate secondary HRP‐conjugated antibody for 1 h at room temperature. The primary and secondary antibodies are listed in Table S8 of the Supporting Information. The protein bands were visualized with ECL (Millipore, Billerica, MA, USA) and a Tanon‐5200 chemical luminescence developing system (Tanon, Shanghai, China). The grayscale values of protein bands were quantified with ImageJ 1.52v, and then all protein expression levels were normalized relative to each sample's *β*‐ACTIN protein level.

### Statistical Analysis

Data were tested for homogeneity (using Bartlett's unequal variances test) and normality (using a Shapiro‐Wilks W‐test). Data were expressed as mean ± standard deviation (SD). Comparisons between multiple exposure groups and the corresponding controls in each exposure experiment were conducted in SPSS 22.0 (IBM, Armonk, NY, USA) and Prism 8.0 (GraphPad Software, Inc., San Diego, CA, USA). The DEGs were visualized with the ggtern package in R. A Student's *t*‐test was applied to determine the difference between two groups. Comparisons between multiple exposure groups, and the corresponding controls in each exposure experiment were conducted by one‐way analysis of variance (ANOVA) followed by a Tukey multiple comparison test. A Kruskal‐Wallis rank sum test was used for significance tests of the pathologic grade of aortas. RSM was conducted using Design‐Expert 11 (Stat‐Ease, Inc., Minneapolis, MN, USA) to explore the synergistic effects caused by combination of PS‐NPs and LCACs.^[^ ^105^ ^]^ A 2‐factor, 3‐level factorial design was applied. The concentrations set for each compound were determined based on the preliminary experiments (Figures 4 and 6). These designs each contained seven combinations and three replications of center point runs (Tables S9 and S10, Supporting Information). A complete quadratic model was employed to fit the experimental data and the significance of terms was determined by ANOVA. Two‐side *P* < 0.05 determined statistical significance.

## Conflict of Interest

The authors declare no conflict of interest.

## Author Contributions

B.W., B.L., Y.H., and Z.L. contributed equally to this work. B.W., B.L., Y.H., and Z.L.: conceptualization, methodology, visualization, formal analysis, and writing the original draft; B.Z., J.D., R.Y., H.X., and Y.D.: visualization, data curation; J.X.: methodology, visualization; X.Y., S.I., and G.I.: funding acquisition, project administration, and writing and reviewing; Y.Z.: conceptualization, methodology, visualization, formal analysis, resources, and writing the original draft; Z.H.: conceptualization, resources, funding acquisition, project administration, and writing, reviewing, and editing.

## Supporting information

Supporting Information

## Data Availability

Research data are not shared.
