# Supplementary material for: Long‐Chain Acyl Carnitines Aggravate Polystyrene Nanoplastics‐Induced Atherosclerosis by Upregulating MARCO
Source: Adv Sci (Weinh). 2023 May 5;10(19):2205876. doi: 10.1002/advs.202205876 (PMC10323628; doi:10.1002/advs.202205876)
Supplement: Supplementary file 1 — Supporting Information [file ADVS-10-2205876-s001.pdf]

ADVANCED SCIENCE
Open Access

Supporting Information

for *Adv. Sci.*, DOI 10.1002/advs.202205876

Long-Chain Acyl Carnitines Aggravate Polystyrene Nanoplastics-Induced Atherosclerosis by Upregulating MARCO

Bo Wang, Boxuan Liang, Yuji Huang, Zhiming Li, Bingli Zhang, Jiaxin Du, Rongyi Ye, Hongyi Xian, Yanhong Deng, Jiancheng Xiu, Xingfen Yang, Sahoko Ichihara, Gaku Ichihara, Yizhou Zhong and Zhenlie Huang**

Supporting Information

Table S1

Physical characteristics of PS-NPs.

Group	Dynamic Light Scattering		
	Size (nm)	PDI	Zeta potential (mV)
Distilled water (500 μg/mL)	55.6 ± 0.7	0.060 ± 0.002	-37.5 ± 2.4
In vitro gastrointestinal digestion (Starch + 500 μg/mL PS-NPs)	94.1 ± 0.8	0.745 ± 0.100	-16.4 ± 0.9
In vitro gastrointestinal digestion (BSA+ 500 μg/mL PS-NPs)	78.6 ± 8.2	0.579 ± 0.072	-12.8 ± 1.1
In vitro gastrointestinal digestion (Glyceryl trioleate + 500 μg/mL PS-NPs)	64.8 ± 6.3	0.652 ± 0.037	-29.9 ± 0.5
In vitro gastrointestinal digestion (Starch + Bovine serum albumin + Glyceryl trioleate + 500 μg/mL PS-NPs)	130.3 ± 6.0	0.743 ± 0.061	-25.5 ± 1.3
ApoE$^{-/-}$ mouse stomach contents (250 mg/kg PS-NPs)	222.7 ± 52.6	0.660 ± 0.051	—[a]
ApoE$^{-/-}$ mouse blood (250 mg/kg PS-NPs)	152.7 ± 79.0	0.835 ± 0.105	—

—[a]: Not available. BSA: bovine serum albumin; PDI: polymer dispersity index. *n* = 3 per group.

Table S2

Lipid categories and subclasses detected in lipidomics.

Lipid category	Lipid subclass	Name of subclass
Phospholipids	CL	Cardiolipin
	LPA	Lysophosphatidic acid
	PA	Phosphatidic acid
	LPC	Lysophosphatidylcholine
	PC	Phosphatidylcholine
	LPE	Lysophosphatidylethanolamine
	PE	Phosphatidylethanolamine
	LPG	Lysophosphatidylglycerol
	PG	Phosphatidylglycerol
	LPI	Lysophosphatidylinositol
	PI	Phosphatidylinositol
	PS	Phosphatidylserine
	PIP	Phosphatidylinositol
	PIP3	Phosphatidylinositol
Sphingolipids	Cer	Ceramides
	CerP	Ceramides phosphate
	Hex1Cer	Simple Glc series
	Hex2Cer	Simple Glc series
	Hex3Cer	Simple Glc series
	CerG2GNAc1	Simple Glc series
	CerG3GNAc1	Simple Glc series
	CerG3GNAc2	Simple Glc series
	GM3	Gangliosides
	GM2	Gangliosides
	GD3	Gangliosides
	LSM	Lysosphingomyelin
	phSM	Sphingomyelin(phytosphingosine)
	SM	Sphingomyelin
	SPH	Sphingosine
	SPHP	Sphingosine phosphate
	ST	Sulfatide

Neutral lipids	ChE	Cholesterol Ester
	DG	Diglyceride
	MG	Monoglyceride
	SiE	Sitosterol Ester
	StE	Stigmasterol Ester
	TG	Triglyceride
	ZyE	Zymosterol Ester
Fatty acyl and other lipids	AcCa	Fatty acyl carnitine
	Co	Coenzyme
	FA	Fatty acid
	OAHFA	OAcyl-(gamma-hydroxy)FA
	WE	Wax esters

Table S3

Body weight of mice during 19-week PS-NP exposure.

Week	Dose (mg/kg)			
	0	2.5	25	250
0	23.51 ± 1.23	23.35 ± 1.37	23.64 ± 1.42	24.19 ± 1.60
1	24.30 ± 1.85	24.85 ± 1.64	24.89 ± 1.33	25.08 ± 2.06
2	25.35 ± 1.59	26.05 ± 1.72	26.04 ± 1.38	25.56 ± 2.27
3	25.84 ± 1.70	26.62 ± 1.79	26.86 ± 3.87	27.50 ± 1.82
4	27.53 ± 1.82	27.53 ± 1.84	28.27 ± 1.97	28.21 ±2.19
5	28.19 ± 2.00	28.47 ± 1.93	28.57 ± 2.18	28.92 ± 2.33
6	28.59 ± 2.20	29.12 ± 2.13	29.10 ± 1.99	29.28 ± 2.63
7	29.27 ± 2.35	29.68 ± 2.12	29.96 ± 2.14	29.99 ± 2.37
8	29.56 ± 2.35	30.30 ± 2.31	30.39 ± 2.26	30.26 ± 2.51
9	29.81 ± 2.43	30.52 ± 2.25	31.05 ±2.11	30.24 ± 2.41
10	30.71 ± 2.37	30.69 ± 2.51	31.43 ± 2.24	31.80 ± 2.32
11	30.79 ± 2.41	31.20 ± 2.33	31.84 ± 2.20	31.25 ± 2.88
12	31.28 ± 2.69	31.23 ± 2.73	32.24 ± 2.50	32.30 ± 2.37
13	31.27 ± 2.70	31.44 ± 2.51	32.66 ± 2.63 *	32.72 ± 2.47 *
14	30.91 ±3.57	32.31 ± 2.82	33.23 ± 2.62 *	33.15 ± 2.78 *
15	31.78 ± 2.69	32.45 ± 2.93	33.44 ± 2.77 *	34.11 ± 2.91 *
16	32.51 ± 3.09	32.73 ± 3.15	33.85 ± 3.11	34.38 ±3.16 *
17	33.11 ± 3.02	33.12 ± 3.45	34.03 ± 3.10	34.68 ±3.61 *
18	32.41 ± 3.25	33.38 ± 3.44	34.29 ±3.18 *	34.84 ±3.71 *
19	33.16 ± 3.11	33.45 ± 3.72	34.04 ± 2.88 *	35.16 ± 3.95 *

n = 25 per group. *, $P < 0.05$, compared with the control. Comparisons were made with ANOVA, followed by Tukey’s multiple comparison tests. ANOVA, analysis of variance.

Table S4

List of antibodies for IF.

Primary antibody	Manufacturer	Catalog	Dilution	Second antibody	Manufacturer	Catalog	Dilution
CD68	Immunoway	YM3050	1:100	FITC conjugated anti-mouse	Servicebio	GB22403	1:200
MARCO	Abcam	Ab239369	1:100	FITC conjugated anti-rabbit	Servicebio	GB21403	1:200

FITC: fluorescein isothiocyanate; IF: immunofluorescence.

Table S5

Information of lipid isotope internal standards.

Compound name	Molecular weight	Exact mass	Chemical fomula	Concentration (μg/mL)
15:0-18:1 (d7) PC	753.11	752.61	$C_{41}H_{73}D7NO_8P$	150.6
15:0-18:1(d7) PE	711.03	710.56	$C_{38}H_{67}D_7NO_8P$	5.3
15:0-18:1(d7) PS (Na Salt)	777.02	776.53	$C_{39}H_{66}D_7NNaO_{10}P$	3.9
15:0-18:1(d7) PG (Na Salt)	764.02	763.54	$C_{39}H_{67}D_7NaO_{10}P$	26.7
15:0-18:1(d7) PI (NH4 Salt)	847.13	846.60	$C_{42}H_{75}D_7NO_{13}P$	8.5
15:0-18:1(d7) PA (Na Salt)	689.94	689.50	$C_{36}H_{61}D_7NaO_8P$	6.9
18:1(d7) Lyso PC	528.72	528.39	$C_{26}H_{45}D_7NO_7P$	23.8
18:1(d7) Lyso PE	486.64	486.35	$C_{23}H_{39}D_7NO_7P$	4.9
18:1(d7) Chol Ester	658.16	657.64	$C_{45}H_{71}D_7O_2$	329.1
18:1(d7) MAG	363.59	363.34	$C_{21}H_{33}D_7O_4$	1.8
15:0-18:1(d7) DAG	587.98	587.55	$C_{36}H_{61}D_7O_5$	8.8
15:0-18:1(d7)-15:0 TAG	812.37	811.77	$C_{51}H_{89}D_7O_6$	52.8
d18:1-18:1(d9) SM	738.12	737.64	$C_{41}H_{72}D_9N_2O_6P$	29.6
Cholesterol (d7)	393.71	393.40	$C_{27}H_{39}D_7O$	98.4

Information was provided in the Certificate of Analysis of SPLASH LipidoMIX™ Internal Standard (Product Number 330707-1EA, Avanti).

Table S6

Information of siRNA.

Primer	Forward primer sequence (5’-3’)	Reverse primer sequence (5'-3')
Si-*Marco*-1	GCUGAAGUUUACUAUAACATT	UGUUAUAGUAAACUUCAGCTT
Si-*Marco*-2	GCAAUGGAUCACUAGCUAUTT	AUAGCUAGUGAUCCAUUGCTT

Table S7

Primer sequences for qPCR.

Gene	GenBank accession	Forward primer (5'-3')	Reverse primer (5'-3')
Cpt1a	NM_013495	CTCCGCCTGAGCCATGAAG	CACCAGTGATGATGCCATTCT
Cpt2	NM_009949	CAGCACAGCATCGTACCCA	TCCCAATGCCGTTCTCAAAAT
Cac	NM_020520	GACGAGCCGAAACCCATCAG	AGTCGGACCTTGACCGTGT
Lcad	NM_007381	TCTTTTCCTCGGAGCATGACA	GACCTCTCTACTCACTTCTCCAG
Marco	NM_010766	GGGTCAAAAAGGCGAATCTTTC	CCCTCTGGAGTAACCGAGCA
β-actin	NM_007393	GGCTGTATTCCCCTCCATCG	CCAGTTGGTAACAATGCCATGT

qPCR: quantitative polymerase chain reaction.

Table S8

List of antibodies for WB.

Primary antibody	Manufacturer	Catalog	Dilution	Second antibody	Manufacturer	Catalog	Dilution
CPT2	Proteintech	AG24897	1:1000	Anti-rabbit	CST	#7074S	1:5000
MARCO	Abcam	Ab239369	1:1000	Anti-mouse	CST	#7076S	1:5000
β-ACTIN	Sigma	A1978	1:5000	Anti-mouse	CST	#7076S	1:5000

Table S9

Concentrations of LCAC 16 and PS-NPs for a 2-factor-3-level factorial design and response in term of the intracellular TC content.

Design point	Factor 1: LCAC 16 (μg/mL)	Factor 2: PS-NPs (μg/mL)	Response 1: TC (nmol/mg)
1	0	0	79.4258
2	0	100	81.5264
3	0	500	148.8760
4	2.5	0	82.8177
5	2.5	100	89.5673
6	2.5	100	87.8380
7	2.5	100	90.7768
8	2.5	100	87.8380
9	5	0	89.7442
10	5	100	128.1800
11	5	500	223.7390

LCAC: long-chain acyl carnitine; TC: total cholesterol.

Table S10

Concentrations of LCAC 18 and PS-NPs for a 2-factor-3-level factorial design and response in term of the intracellular TC content.

Design point	Factor 1: LCAC 18 (μg/mL)	Factor 2: PS-NPs (μg/mL)	Response 1: TC (nmol/mg)
1	0	0	79.4258
2	0	100	81.5264
3	0	500	148.876
4	2.5	0	77.7173
5	2.5	100	89.072
6	2.5	100	87.638
7	2.5	100	87.9984
8	2.5	500	161.117
9	5	0	92.6929
10	5	100	127.79
11	5	500	216.53

LCAC: long-chain acyl carnitine; TC: total cholesterol.

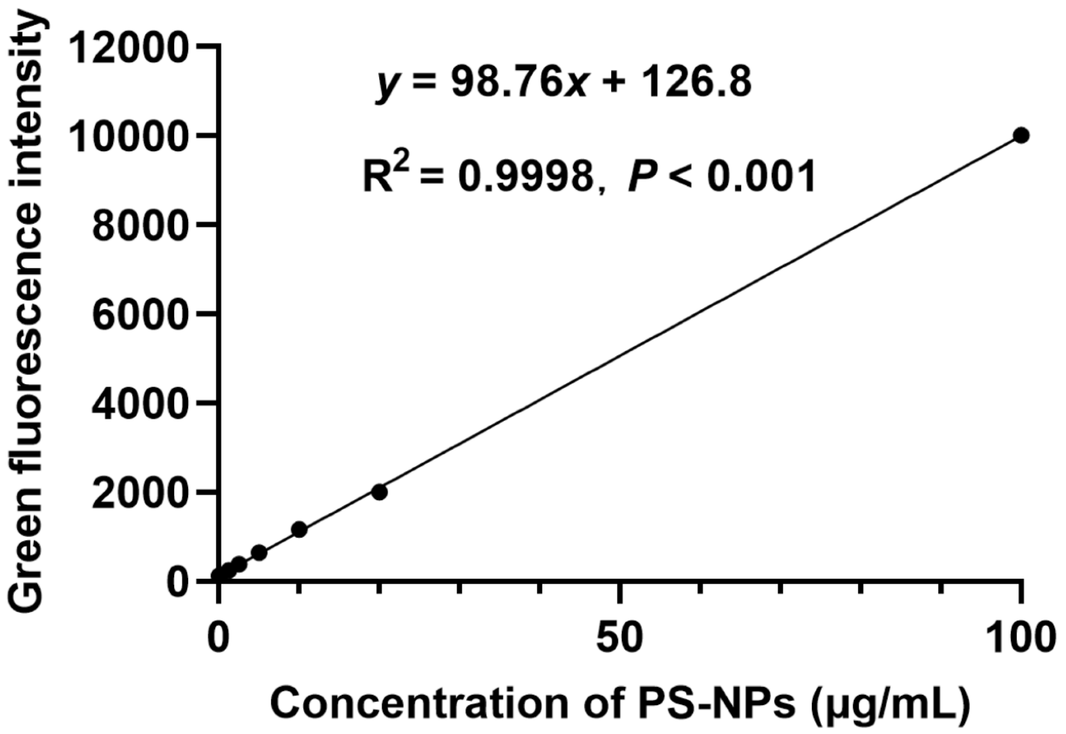

Figure S1. The standard curve of green fluorescence PS-NP concentration for blood. It indicates the green fluorescence intensity of PS-NP particles was linearly correlated with its concentrations.

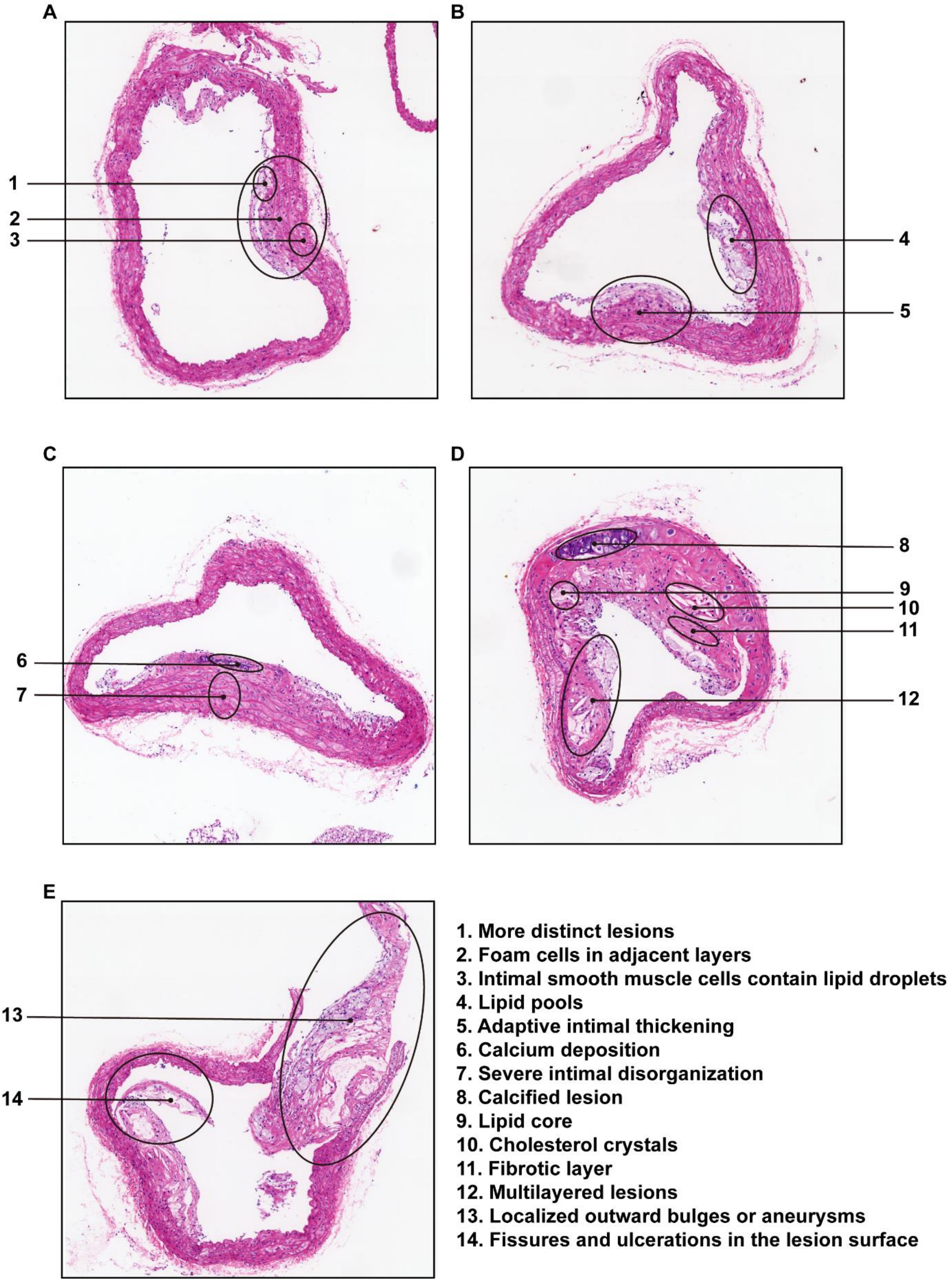

Figure S2. The pathological grades of aortic atherosclerosis. A) grade Ⅱ, B) grade Ⅲ, C) grade Ⅳ, D) grade Ⅴ, and E) grade Ⅵ.

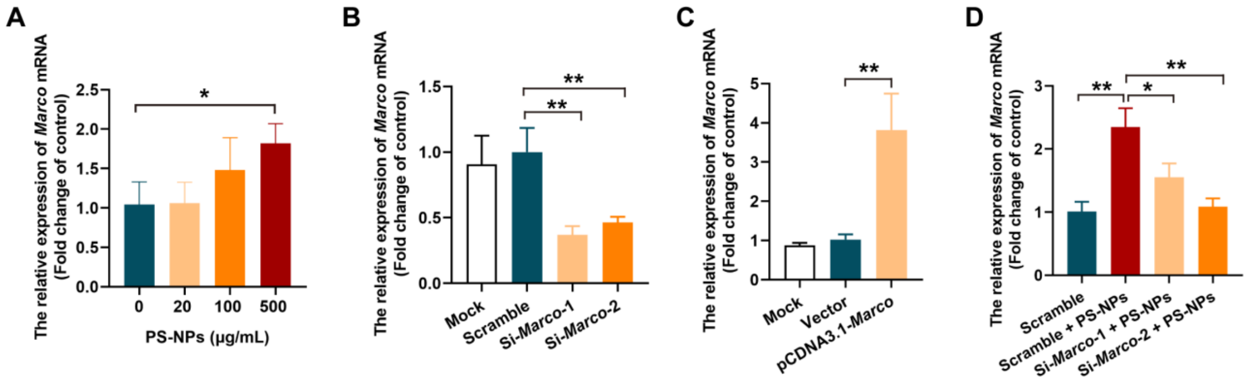

Figure S3. The *Marco* mRNA expression responded to various treatments *in vitro*. A) PS-NPs enhanced *Marco* mRNA expression in the RAW264.7 cells. B) *Marco* mRNA knock-down by si-*Marco*-1 and si-*Marco*-2. C) *Marco* mRNA overexpression by transfecting a pCDNA3.1-*Marco* plasmid. D) MARCO knock-down reversed the PS-NP-exacerbated *Marco* mRNA expression when the foam cells were transfected with si-*Marco* before treatment with PS-NPs.

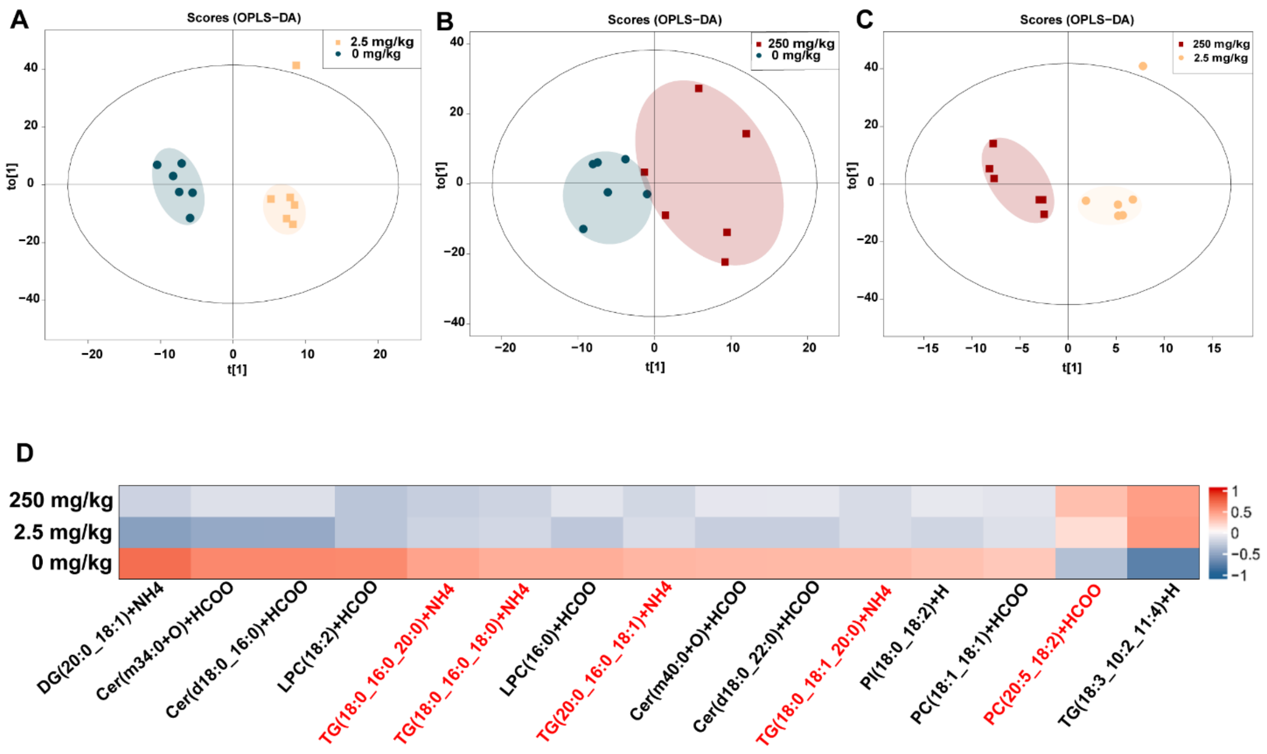

Figure S4. OPLS-DA separated the groups based on PS-NP exposure. A) Samples from the 2.5 mg/kg PS-NP group and the control group were well separated, with an outlier in the 2.5 mg/kg group. B) Larger within-group variation at a dose of 250 mg/kg, compared with the control group. C) Samples from 2.5 and 250 mg/kg PS-NP groups were well separated, with an outlier in the 2.5 mg/kg group. OPLS-DA: Orthogonal Partial Least Squares Discriminant Analysis. D) Common differentially expressed lipid profiles in the 2.5 and 250 mg/kg PS-NP groups. The change of common differentially expressed lipids in a dose-dependent manner marked with red color.

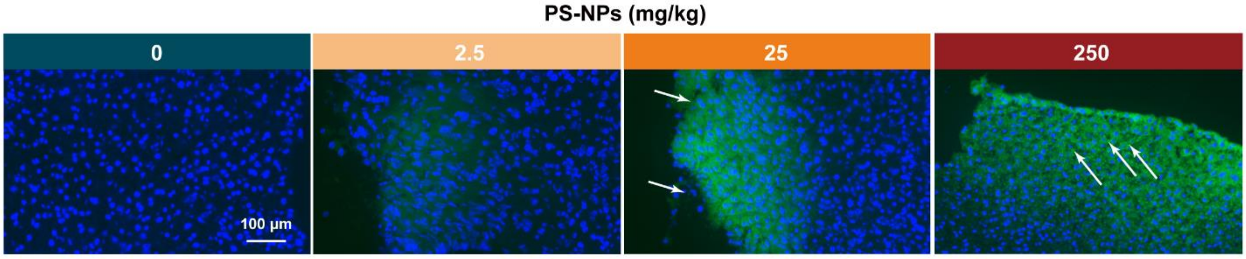

Figure S5. PS-NP particles dose-dependently distributed in the mouse liver in the *ApoE*$^{-/-}$ mice after 24 h PS-NP exposure. White arrows showed PS-NP particles in the liver tissues.

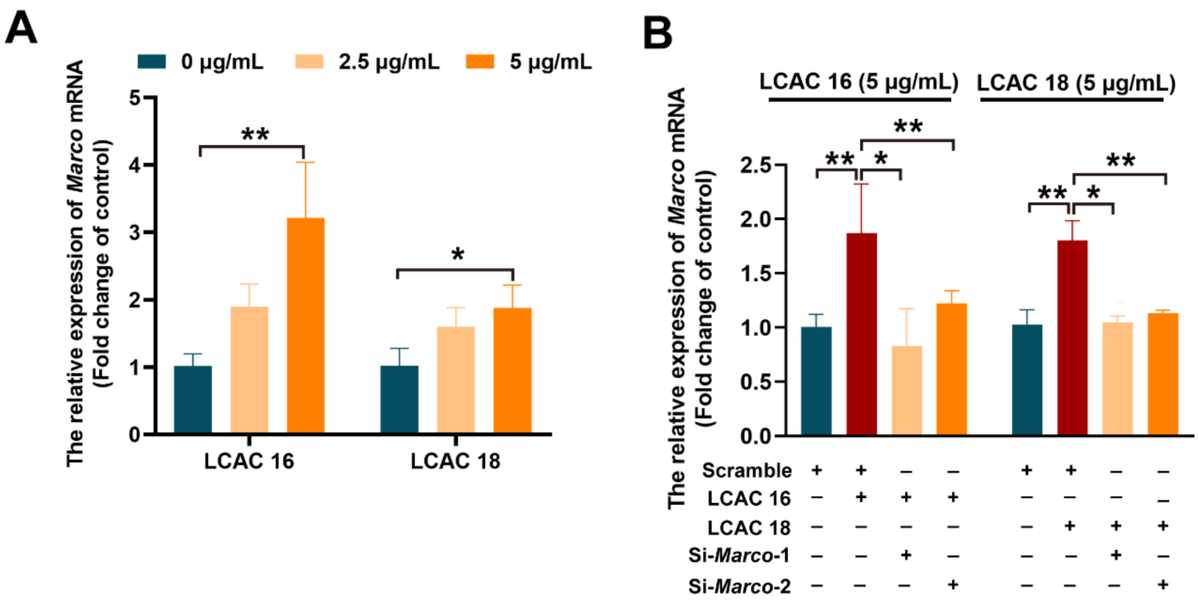

Figure S6. A) Both of LCAC 16 and LCAC 18 at 5 μg/mL increased *MARCO* mRNA levels. B) MARCO knock-down reversed the LCAC 16- and LCAC 18-enhanced *MARCO* mRNA levels were transfected with si-MARCO before LCAC 16 and LCAC 18 treatments.
